# ERAD components Derlin-1 and Derlin-2 are essential for postnatal brain development and motor function

**DOI:** 10.1016/j.isci.2021.102758

**Published:** 2021-06-19

**Authors:** Takashi Sugiyama, Naoya Murao, Hisae Kadowaki, Keizo Takao, Tsuyoshi Miyakawa, Yosuke Matsushita, Toyomasa Katagiri, Akira Futatsugi, Yohei Shinmyo, Hiroshi Kawasaki, Juro Sakai, Kazutaka Shiomi, Masamitsu Nakazato, Kohsuke Takeda, Katsuhiko Mikoshiba, Hidde L. Ploegh, Hidenori Ichijo, Hideki Nishitoh

**Affiliations:** 1 Laboratory of Biochemistry and Molecular Biology, Department of Medical Sciences, University of Miyazaki, 5200 Kihara, Kiyotake, Miyazaki 889-1692, Japan; 2 Department of Behavioral Physiology, Faculty of Medicine, University of Toyama, Toyama 930-0194, Japan; 3 Research Center for Idling Brain Science, University of Toyama, Toyama, Japan; 4 Section of Behavioral Patterns, Center for Genetic Analysis of Behavior, National Institute for Physiological Sciences, Japan; 5 Division of Systems Medical Science, Institute for Comprehensive Medical Science, Fujita Health University, Toyoake, Aichi 470-1192, Japan; 6 Division of Genome Medicine, Institute for Genome Research, Tokushima University, Tokushima 770-8503, Japan; 7 Department of Basic Medical Sciences, Kobe City College of Nursing, 3-4 Gakuen-nishi-machi, Nishi-ku, Kobe 651-2103, Japan; 8 Department of Medical Neuroscience, Graduate School of Medical Sciences, Kanazawa University, Kanazawa 920-8640, Japan; 9 Division of Metabolic Medicine, Research Center for Advanced Science and Technology, The University of Tokyo, Tokyo 153-8904, Japan; 10 Division of Molecular Physiology and Metabolism, Tohoku University Graduate School of Medicine, Sendai 980-8574, Japan; 11 Division of Neurology, Respirology, Endocrinology, and Metabolism, Department of Internal Medicine, Faculty of Medicine, University of Miyazaki, 5200 Kihara, Kiyotake, Miyazaki 889-1692, Japan; 12 Department of Cell Regulation, Graduate School of Biomedical Sciences, Nagasaki University, 1-14 Bunkyo-machi, Nagasaki 852-8521, Japan; 13 RIKEN Center for Life Science Technologies (CLST), Minatojima-minamimachi, Chuo-ku, Kobe 650-0047, Japan; 14 Shanghai Institute for Advanced Immunochemical Studies (SIAIS), Shanghai Tech University, Shanghai, China; 15 Department of Biomolecular Science, Faculty of Science, Toho University, Funabashi, Japan; 16 Boston Children's Hospital and Harvard Medical School, 1 Blackfan Circle, Boston, MA 02115, USA; 17 Laboratory of Cell Signaling, Graduate School of Pharmaceutical Sciences, The University of Tokyo, Tokyo 113-0033, Japan; 18 Frontier Science Research Center, University of Miyazaki, 5200 Kihara, Kiyotake, Miyazaki 889-1692, Japan

**Keywords:** Biological sciences, Neuroscience, Molecular neuroscience

## Abstract

Derlin family members (Derlins) are primarily known as components of the endoplasmic reticulum-associated degradation pathway that eliminates misfolded proteins. Here we report a function of Derlins in the brain development. Deletion of *Derlin-1* or *Derlin-2* in the central nervous system of mice impaired postnatal brain development, particularly of the cerebellum and striatum, and induced motor control deficits. Derlin-1 or Derlin-2 deficiency reduced neurite outgrowth *in vitro* and *in vivo* and surprisingly also inhibited sterol regulatory element binding protein 2 (SREBP-2)-mediated brain cholesterol biosynthesis. In addition, reduced neurite outgrowth due to Derlin-1 deficiency was rescued by SREBP-2 pathway activation. Overall, our findings demonstrate that Derlins sustain brain cholesterol biosynthesis, which is essential for appropriate postnatal brain development and function.

## Highlights

• Derlin-1 and Derlin-2 are essential for postnatal brain development and function
• Chemical chaperon does not ameliorate the phenotype of Derlin-deficient neuron
• Derlin regulates SREBP-2 activation and promotes brain cholesterol biosynthesis
• Derlin-mediated cholesterol biosynthesis is essential for neurite outgrowth

## Graphical abstract

**Figure:**
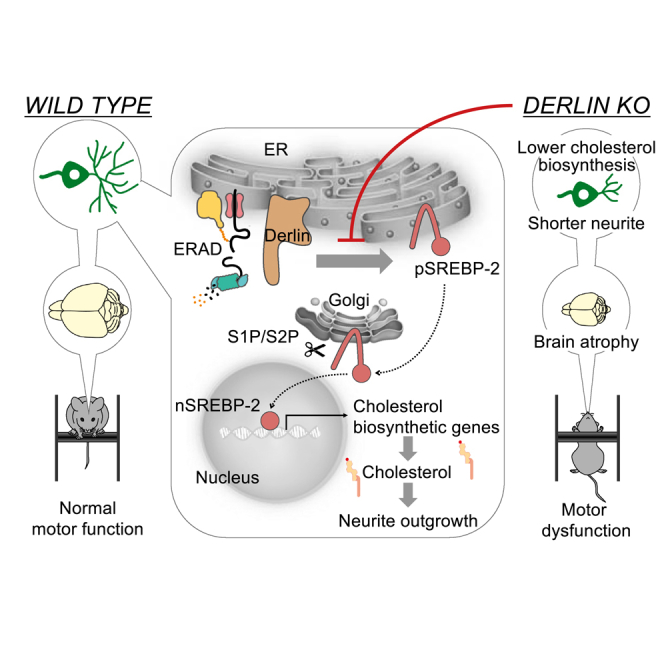

## Introduction

To prevent misfolding of proteins and ensuing pathological endoplasmic reticulum (ER) stress, cells activate the unfold protein response (UPR), which restores ER protein homeostasis by refolding or degrading unfolded proteins. The Derlin family members, Derlin-1, Derlin-2, and Derlin-3, are ER membrane proteins that associate with various other ER proteins, such as Sel1L, Hrd1, Herp, and p97, to form the ER-associated degradation (ERAD) complex, which eliminates unfolded proteins (Christianson et al., 2008; Lilley and Ploegh, 2004; Ye et al., 2004). In addition, Derlins contribute to ER protein quality control by facilitating the degradation of newly synthesized ER-targeted proteins, termed ER stress-induced pre-emptive quality control (ERpQC) (Kadowaki et al., 2015, 2018). In rodents, *Derlin-1* and *Derlin-2* mRNAs are ubiquitously expressed, including throughout the central nervous system (CNS), whereas *Derlin-3* mRNA expression is restricted to specific tissues except the brain (Oda et al., 2006). Whole-body deletion of Derlin-1 causes lethality at embryonic day 7 (E7) to E8 (Eura et al., 2012), whereas most Derlin-2-deficient mice demonstrate perinatal lethality due to feeding failure, resulting in only 4% survival at weaning (Dougan et al., 2011). Unlike Derlin-1- or Derlin-2-deficient mice, Derlin-3-deficient mice are normally born and grow as well as wild-type mice (Eura et al., 2012). Therefore, Derlin-1 and Derlin-2 appear to be essential during ontogeny. We have reported that the interactions of Derlin-1 with amyotrophic lateral sclerosis-related superoxide dismutase 1 (SOD1) mutants trigger a pathological UPR, leading to motor neuron dysfunction (Nishitoh et al., 2008). However, the contributions of Derlin-1 to normal brain development have not been established.

Cellular cholesterol level is tightly controlled by transcriptional as well as posttranscriptional regulation of biosynthetic enzymes (Luo et al., 2020). Genes encoding many cholesterol biosynthetic enzymes are induced by activation of the ER membrane-anchored transcription factor sterol regulatory element binding protein 2 (SREBP-2). Under cellular cholesterol depletion, the ER transmembrane protein Scap escorts SREBP-2 from the ER to the Golgi apparatus, where it is sequentially cleaved by the Golgi-resident site-1 and site-2 proteases. Following cleavage, the amino-terminal form of SREBP-2 translocates to the nucleus, where it induces the transcription of cholesterol biosynthesis-related genes. In contrast, when excess cholesterol accumulates in the ER membrane, Scap interacts with the insulin-induced gene (Insig)-1 and Insig-2, resulting in the inhibition of Scap/SREBP-2 transport to the Golgi apparatus. In addition, the ERAD-related-E3 ubiquitin ligases RNF145, gp78, Hrd1, and MARCH6 ubiquitinate and degrade the cholesterol biosynthetic enzymes (van den Boomen et al., 2020). Another ER-resident E3 ligase TRC8 interacts with the Scap/SREBP-2 complex, which in turn inhibits cholesterol biosynthesis (Irisawa et al., 2009). Insigs are ubiquitinated by TRC8 and gp78 and degraded by the ERAD pathway only when unbound from Scap (Liu et al., 2012; Luo et al., 2020). Hence, it is still uncertain whether the ERAD pathway positively or negatively regulates cholesterol biosynthesis, including in the brain, which contains ∼20%–25% of all cholesterol in the body (Dietschy and Turley, 2004).

In the present study, we generated CNS-specific Derlin-1- or Derlin-2-deficient mice and investigated changes in brain development and function. Both mice models exhibited widespread postnatal brain atrophy, which was particularly severe in the cerebellum and striatum, as well as reduced neurite outgrowth and motor function deficits. Both Derlin-1 and Derlin-2 deficiency, surprisingly, also suppressed SREBP-2-mediated cholesterol biosynthesis in the cerebellum, and activation of the SREBP-2 pathway rescued neurite outgrowth from Derlin-1-deficient neurons. Altogether, these findings illustrate that Derlins are indispensable for postnatal brain development and function by sustaining the SREBP-2-mediated cholesterol biosynthetic pathway.

## Results

### Developmental defects in mouse brain due to deletion of *Derlin-1* or *Derlin-2*

**Figure 1:**
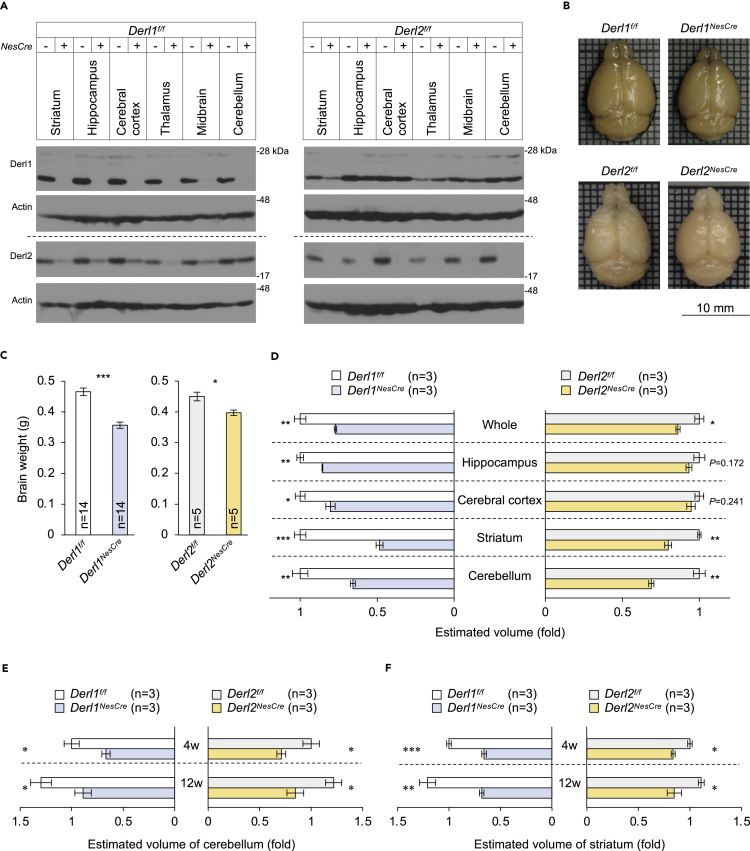
Developmental defects in the brains of Derlin-1- and Derlin-2-deficient mice (A) Expression of Derlin-1 and Derlin-2 in the brains of *Derl1*^*NesCre*^ and *Derl2*^*NesCre*^ mice at 4 weeks of age. Tissue extracts were analyzed by immunoblotting (IB) with the indicated antibodies. *NesCre* (+), *Derl1*^*NesCre*^ or *Derl2*^*NesCre*^; *NesCre* (−), *Derl1*^*f/f*^ or *Derl2*^*f/f*^. (B) Representative gross brain images of mice aged 37 weeks. (C) Brain weights of mice aged 37 weeks. (D) Volumetric analysis of Derlin-deficient and control brains aged 37 weeks. Regional volumes were estimated according to the Cavalieri's principle using manually measured cross-sectional areas. (E and F) Age-dependent volumetric analysis of the cerebellum (E) and striatum (F). Bar graphs are presented as mean ± SEM. ∗P < 0.05, ∗∗P < 0.01, and ∗∗∗P < 0.001 by Student's t test. n indicates the number of animals. See also Figure S1.

Derlin-1 and Derlin-2 are widely expressed in the mature CNS (Figure 1A), suggesting essential roles in physiological brain functions. We established CNS-specific deletion mutants by crossing mice harboring *Derl1* and *Derl2* genes flanked by *loxP* sites (*Derl1*^*f/f*^ and *Derl2*^*f/f*^) with mice expressing Cre recombinase driven by the *nestin* promoter [*Tg(Nes-Cre)1Kag* mice] (Dougan et al., 2011; Isaka et al., 1999) (Figures S1A–S1D). The resulting *Derl1*^*f/f*^*;Tg(Nes-Cre)1Kag* (*Derl1*^*NesCre*^) and *Derl2*^*f/f*^*;Tg(Nes-Cre)1Kag* (*Derl2*^*NesCre*^) mice exhibited markedly reduced expression levels of Derlin-1 and Derlin-2 in the striatum, hippocampus, cerebral cortex, thalamus, midbrain, and cerebellum (Figure 1A). In addition, Derlin-1 deletion reduced Derlin-2 expression in certain brain regions (Figure 1A), consistent with previous findings that Derlin-1 supports the expression of Derlin-2 (Dougan et al., 2011; Kadowaki et al., 2015). The gross structure of the brain on postnatal day 0 (P0) appeared normal in both *Derl1*^*NesCre*^ mice (Figures S1E and S1F) and systemic *Derl2* deletion mice (*Derl2*^*−/−*^) (Dougan et al., 2011); however, *Derl1*^*NesCre*^ and *Derl2*^*NesCre*^ mice demonstrated substantial microcephaly with significant reductions in brain size, weight, and whole volume at 37 weeks of age (Figures 1B–1D). Serial sections through whole brains of these mice aged 37 weeks revealed particularly dramatic volume loss in cerebellum and striatum (Figure 1D). Atrophy of the cerebellum and striatum was observed in both *Derl1*^*NesCre*^ and *Derl2*^*NesCre*^ mice as early as 4 weeks of age and increased progressively up to 12 weeks of age compared with age-matched *Derl1*^*f/f*^ and *Derl2*^*f/f*^ control mice (Figures 1E and 1F). Derlin-1- or Derln-2-deficient mice exhibited both lower brain and body weight compared with the control (Figures S1G and S1H). The brain and body weight correlated with each other in the *Derl1*^*NesCre*^ but not in the *Derl2*^*NesCre*^ mice (Figures S1I and S1J). Although we cannot exclude the possibility that brain atrophy depends on body growth inhibition, there might be another mechanism by which CNS-specific Derlin-1 or Derlin-2 deficiency induces brain atrophy. Taken together, Derlin-1 and Derlin-2 are both critical for postnatal brain development, particularly for normal growth of the cerebellum and striatum, whereas gross embryonic development appears to progress normally in the absence of these proteins.

### Requirement of Derlin-1 and Derlin-2 for neurite outgrowth

**Figure 2:**
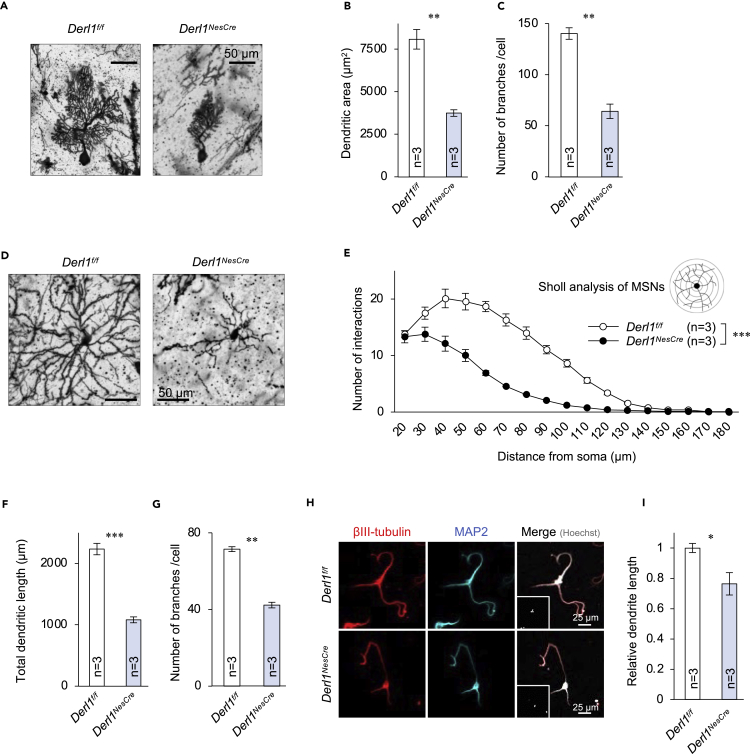
Requirement of Derlin-1 for neurite outgrowth (A–C) Morphological analysis of cerebellar Purkinje cells at 30 weeks of age. (A) Representative images of Golgi-stained Purkinje cells, (B) quantification of the dendritic area, and (C) the number of branches. The dendritic area was measured using ImageJ software. Ten Purkinje cells were measured in each mouse, and the average from three unrelated mice per genotype are presented. (D–G) Morphological analysis of striatal MSNs at 30 weeks of age. (D) Representative images of Golgi-stained MSNs. (E) Sholl analysis showing the number of dendritic intersections at the indicated distances from the soma. (F) Quantification of total dendritic length and (G) number of branches. Ten MSNs were measured from each mouse, and the average from three unrelated mice per genotype is presented. (H and I) Morphological analysis of primary cultured cortical neurons derived from mouse embryos at 6 days *in vitro*. (H) Representative immunofluorescence images of βIII-tubulin- and MAP2-positive neurons. (I) The dendritic length was measured using ImageJ software. More than 25 primary cultured neurons from each embryo were measured, and the average from three unrelated embryos per genotype are presented. Data are presented as mean ± SEM. ∗P < 0.05, ∗∗P < 0.01, and ∗∗∗P < 0.001 by Student's t test (B, C, F, G, and I) or repeated measures ANOVA (E). See also Figures S2 and S3.

Immunohistological staining using an anti-NeuN antibody was conducted to demonstrate the extent of cerebellar atrophy in *Derl1*^*NesCre*^ and *Derl2*^*NesCre*^ mice (Figure S2A). Quantitative analysis of the entire cerebellum revealed developmental defects in the molecular layer containing dendrites of Purkinje cells (Figure S2B). In several neurodegenerative disorders (e.g., spinocerebellar ataxia), the number of Purkinje cells is reduced owing to enhanced death rate, leading to cerebellar atrophy and functional deficits (Koeppen, 1998). In contrast, the number of calbindin-positive Purkinje cells per unit length of the cell body layer was not reduced in *Derl1*^*NesCre*^ and *Derl2*^*NesCre*^ mice compared with corresponding *Derl1*^*f/f*^ and *Derl2*^*f/f*^ control mice (Figures S2C and S2D). Similarly, the number of NeuN-positive neurons per unit area of striatum was not affected by Derlin-1 or Derlin-2 deficiency (Figures S2E and S2F). Hence, we hypothesized that Derlin-1 and Derlin-2 deficiencies may induce regional atrophy by suppressing the morphological maturation of individual neurons rather than by reducing cell number. We then investigated possible failure of morphological maturation by Purkinje cells and striatal medium spiny neurons using Golgi staining. Consistent with immunohistochemistry (Figure S2A), Golgi-stained Purkinje cells demonstrated smaller dendritic areas and branch numbers in both *Derl1*^*NesCre*^ mice (Figures 2A–2C) and *Derl2*^*NesCre*^ mice (Figures S2G–S2I). Sholl analysis, which measures branching along the dendritic length, also revealed morphological abnormalities of striatal MSN dendrites in *Derl1*^*NesCre*^ mice (Figures 2D–2G) and *Derl2*^*NesCre*^ mice (Figures S2J–S2M). In contrast, neither Derlin-1 nor Derlin-2 deficiency affected the dendritic arbor complexity as indicated by the number of branches per unit area of Purkinje dendritic field and per unit length of striatal MSN dendrite (Figures S2N–S2Q). Thus, Derlin deficiency appears to induce regional atrophy by reducing the area and volume of individual dendritic fields. Together, these results also suggest that Derlin-1 and Derlin-2 are required for proper neurite outgrowth.

Derlin-1 deficiency-induced atrophy was also observed in the cerebral cortex (Figure 1D), which provides a convenient source of embryonic neurons to examine the effects of Derlins on neurite length more precisely under controlled *in vitro* conditions. Indeed, cultured primary cortical neurons derived from Derlin-1-deficient mice also exhibited shorter neurites compared with neurons derived from *Derl1*^*f/f*^ mice (Figures 2H and 2I). This *in vitro* primary neuron culture contained only neurons without other cell types, such as astrocytes and microglia, suggesting that Derlin-1 might be required for a cell-autonomous dendritic outgrowth. Although Derlin-1-deficient neurons could potentially exhibit a delay in the dendritic outgrowth, our results further demonstrate that Derlin-1 promotes the elongation of dendrites, a process necessary for postnatal development of functional neural circuits.

**Figure 3:**
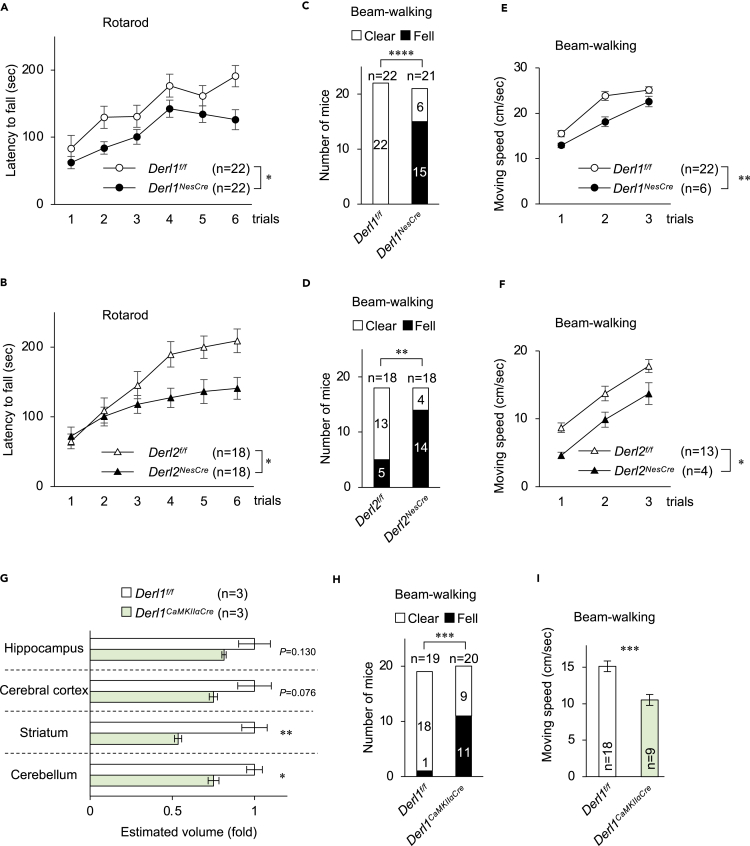
Motor dysfunction of CNS-specific Derlin-1- and Derlin-2-deficient mice (A and B) Rotarod test performance at 11–12 weeks of age. Each mouse performed three trials per day for a total of six trials over 2 days. (C–F) Beam-walking performance at 32 weeks of age (C and E) and 26–27 weeks of age (D and F). Shown are the number of mice that fell off one or more times of three trials (Fell) and the number that safely reached the platform three of three trials (Clear). The time required to walk across the beam was also recorded to calculate movement speed. (G) Volumetric analysis of brain regions at 12 weeks of age. Volumes were estimated as described in Figure 1D. (H and I) Beam-walking performance at 24 weeks of age. The number of mice that fell off and speed of crossing were measured as described in *C*–*F*. Data are presented as mean ± SEM. ∗P < 0.05, ∗∗P < 0.01, ∗∗∗P < 0.001, and ∗∗∗∗P < 0.0001 by Student's t test (G and I), Fisher's exact test (C, D, and H), and repeated measures ANOVA (A, B, E, and F). n indicates the number of animals. See also Figure S4.

Many degenerative diseases are associated with neuroinflammation as evidenced by the appearance of reactive astrocytes and activated microglia in atrophied brain regions. The cerebellum and striatum of *Derl1*^*NesCre*^ mice exhibited more S100β- and glial fibrillary acidic protein (GFAP)-double-positive (immunoreactive) astrocytes and Iba1-positive (active) microglia than those of the control (Figures S3A–S3F). However, *Derl2*^*NesCre*^ mice did not exhibit enhanced numbers of reactive astrocytes and microglia in the cerebellum or striatum (Figures S3G–S3L); they demonstrated both regional brain atrophy (Figure 1D) and functional motor deficits (see Figure 3). Hence, neuroinflammation does not appear necessary for the observed abnormalities in regional brain structure.

### Motor dysfunction due to Derlin-1 or Derlin-2 deficiency in the brain

Neural circuits encompassing striatal and cerebellar neurons are critical mediators of motor control; therefore, regional atrophy may impair performance on motor function tests. In the rotarod test, *Derl1*^*NesCre*^ and *Derl2*^*NesCre*^ mice demonstrated significantly shorter latencies to lose balance and fall off compared with *Derl1*^*f/f*^ and *Derl2*^*f/f*^ control mice (Figures 3A and 3B), suggesting impaired motor coordination. A previous study reported that rotarod performance is negatively correlated with body weight (McFadyen et al., 2003), which may account for performance differences among some transgenic lines. However, *Derl1*^*NesCre*^ and *Derl2*^*NesCre*^ mice were lighter than control mice in body weight (Figures S1G and S1H); hence, the reduction in rotarod performance was likely weight independent.

To further assess motor coordination deficits, we conducted beam-walking tests in which mice were required to traverse an elongated cylindrical rod. Both *Derl1*^*NesCre*^ and *Derl2*^*NesCre*^ mice fell off more frequently than corresponding control mice (Figures 3C and 3D), and *Derl1*^*NesCre*^ and *Derl2*^*NesCre*^ mice moved more slowly across the rod than control mice during all three trials (Figures 3E and 3F). Together, these results indicate that neuron-specific Derlin-1 or Derlin-2 deficiency impairs motor function and coordination. However, Cre recombinase driven by the *nestin* promoter also deletes the target genes flanked by *loxP* sites in astrocytes and oligodendrocytes. To examine the unique contributions of striatal and cerebellar neurons to these functional deficits, we established neuron-specific deletion mutants by crossing *Derl1*^*f/f*^ mice with mice expressing Cre recombinase driven by the *CaMKIIα* promoter (Karpati et al., 2019). The *Derl1*^*f/f*^*;C57BL/6-TgN(a-CaMKII-nlCre)/10* (*Derl1*^*CaMKIIαCre*^) mice exhibited reduced Derlin-1 expression levels markedly in the striatum, hippocampus, cerebral cortex, thalamus, and midbrain, and partially in the cerebellum (Figure S4A). Consistent with observations in *Derl1*^*NesCre*^ mice, *Derl1*^*CaMKIIαCre*^ mice also exhibited brain atrophy (Figures S4B and S4C) and significant volume loss in the cerebellum and striatum (Figure 3G). Moreover, *Derl1*^*CaMKIIαCre*^ mice fell off the beam more frequently during the beam-walking test than control mice (Figure 3H) and were significantly slower than control mice (Figure 3I). Of interest, neuroinflammation was observed in the striatum, but not in the cerebellum, of *Derl1*^*CaMKIIαCre*^ mice (Figures S3M and S3N). Together, our findings suggest that neuronal Derlin-1 contributes to brain development and motor function.

### ER stress response in the cerebella of Derlin-1- and Derlin-2-deficient mice

**Figure 4:**
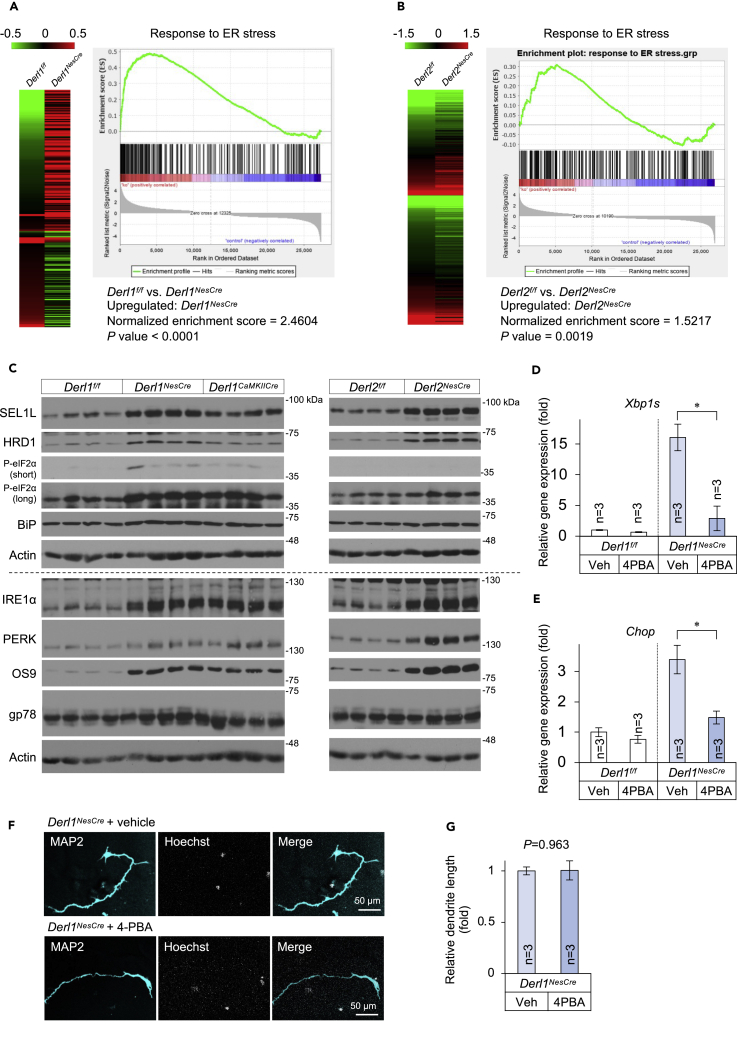
ER stress response in the cerebella of Derlin-1- and Derlin-2-deficient mice (A and B) Heatmap (left) and gene set enrichment analysis (GSEA, right) showing differential expression of 235 genes related to the GO term “Response to ER stress.” Genes upregulated by Derlin deletion are indicated in red and downregulated genes in green. GSEA shows gene expression changes in the cerebellum of *Derl1*^*NesCre*^ or *Derl2*^*NesCre*^ mice relative to control mice. The enrichment plot shows the distribution of genes in the set of “Response to ER stress” that are positively (red) or negatively (blue) correlated with Derlin-1 or Derlin-2 deficiency. (C) Expression levels of ER stress-related molecules in the cerebellum at 4–5 weeks of age. Whole tissue lysates from cerebella of *Derl1*^*f/f*^, *Derl1*^*NesCre*^, *Derl1*^*CaMKIIαCre*^, *Derl2*^*f/f*^, and *Derl2*^*NesCre*^ mice were analyzed by IB with the indicated antibodies. (D–G) A chemical chaperon 4-PBA does not mitigate the reduced neurite outgrowth observed in Derlin-1-deficient neurons. Cortical neurons derived from *Derl1*^*f/f*^ and *Derl1*^*NesCre*^ embryos were treated with vehicle (water) or 1 mM 4-PBA and cultured for 3 days. Gene expression levels of *Xbp1s* and *Chop* in neurons were estimated by qPCR and normalized to that of *S18* (D and E). Neurons were stained with anti-MAP2 antibody (F). Dendritic length of MAP2-positive neuron was quantified using ImageJ software (G). One hundred neurons were measured in each culture dish and averaged to obtain n = 1. Bar graphs are presented as mean ± SEM. ∗P < 0.05 by Student's t test. n indicates independent dishes (D, E, and G). See also Figure S5, Table S1. Gene list whose expression level changed by more than 1.5-fold or less than 0.67-fold at postnatal day 28 in the cerebellum of Derlin-1-deficient mice, related to figure 4, Table S2. Gene list whose expression level changed by more than 1.5-fold or less than 0.67-fold at postnatal day 28 in the cerebellum of Derlin-2-deficient mice, related to figure 4, Table S3. Primers for quantitative real-time PCR, Related to key resources table.

To elucidate the molecular mechanisms by which *Derl1* or *Derl2* deletion reduces neurite outgrowth and disrupts brain development, we compared cerebellar gene expression profiles among *Derl1*^*NesCre*^, *Derl2*^*NesCre*^, and control (*Derl1*^*f/f*^ and *Derl2*^*f/f*^) mice at P28 using DNA microarrays. In cerebella of *Derl1*^*NesCre*^ and *Derl2*^*NesCre*^ mice, expression levels of 4,017 and 1,984 genes were upregulated (>1.5-fold) and 1,854 and 2,015 downregulated (<0.67-fold) compared with control mice, respectively (Tables S1 and S2). In both *Derl1*^*NesCre*^ and *Derl2*^*NesCre*^ mice, 741 genes showed higher than 1.5-fold expression (Figure S5A). Among them, 13 genes were categorized as ER stress-responsive genes by gene ontology (GO) annotation (Figures 4A, 4B, and S5A). To examine whether Derlin-1 or Derlin-2 deficiency activates the UPR pathway, we analyzed *Derl1*^*NesCre*^, *Derl2*^*NesCre*^, and control mice cerebellar total RNA samples using quantitative real-time PCR (qPCR). Derlin-1 or Derlin-2 deficiency increased the spliced *Xbp1* (*Xbp1s*) and *Chop* mRNA levels (Figures S5B and S5C). Moreover, the immunoblotting analysis revealed the increased expression of the ERAD complex, SEL1L, HRD1, and OS9, just as well as the activation of the IRE1α-XBP1 and PERK-eIF2α pathways, suggesting that Derlin deficiency may alter the ERAD function (Figures 4C and S5D–S5K). To investigate whether ER stress could contribute to the dendrite shortening of the Derlin-1-deficient neurons, we treated primary cortical neurons with the chemical chaperon 4-phenylbutyric acid (4-PBA). The 4-PBA treatment significantly mitigated the UPR in the *Derl1*^*NesCre*^ mouse neurons (Figures 4D and 4E). However, surprisingly, the treatment with 4-PBA had no effect on the reduced neurite outgrowth of Derlin-1-deficient neurons (Figures 4F and 4G), suggesting that impaired ER quality control may not contribute to the reduced neurite outgrowth and disrupted brain development observed in *Derl1*^*NesCre*^ and *Derl2*^*NesCre*^ mice.

### Requirement of Derlin-1 and Derlin-2 for cholesterol biosynthesis in the cerebellum

**Figure 5:**
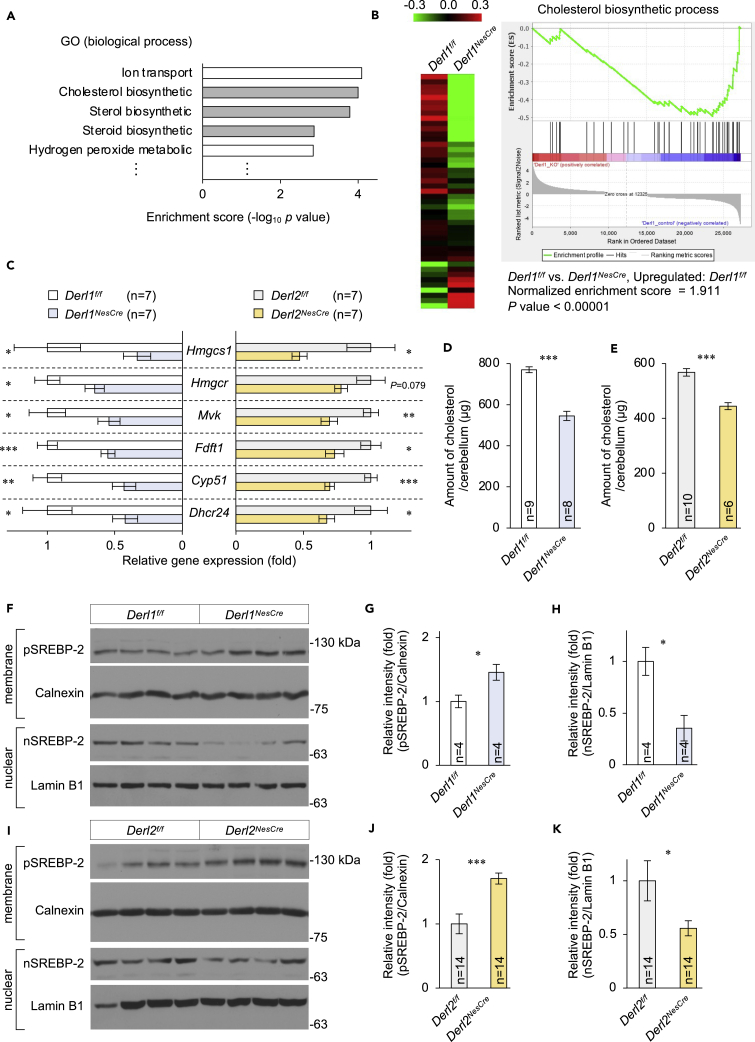
Requirement of Derlin-1 and Derlin-2 for the activation of SREBP-2 pathway (A) Gene ontology (GO) analysis was performed on 1,854 genes showing >0.67-fold lower expression in the cerebellum of *Derl1*^*NesCre*^ mice at P28 compared with age-matched *Derl1*^*f/f*^ mice. The top five GO terms in the biological process category are listed (B) Heatmap (left) and gene set enrichment analysis (GSEA, right) showing differential expression of 45 genes in the cerebellum categorized by the GO term “Cholesterol biosynthetic process.” (C) Expression of cholesterol biosynthesis-related genes in the cerebellum at P28. (D and E) Quantification of total cholesterol in the cerebellum of *Derl1*^*NesCre*^ and *Derl2*^*NesCre*^ mice at 4 weeks of age. (F–K) Expression levels of precursor (pSREBP-2) and nuclear (nSREBP-2) forms of SREBP-2 in the cerebellum at P28. The membrane and nuclear fractions were analyzed by IB using the indicated antibodies. pSREBP-2, nSREBP-2, calnexin, and lamin B1 band intensities were measured from unrelated animals per genotype. Amounts of pSREBP-2 and nSREBP-2 were normalized to those of calnexin and lamin B1, respectively. Bar graphs are presented as mean ± SEM. ∗P < 0.05, ∗∗P < 0.01, and ∗∗∗P < 0.001 by Student's t test. n indicates the number of animals. See also Figure S6, Table S3.

In addition to the ER stress-related genes, we found significant enrichment of cholesterol biosynthesis-related genes among downstream targets of Derlin-1 (Figures 5A and 5B). Efficient cholesterol biosynthesis is essential for neurite elongation because neuronal membranes contain high levels of cholesterol (Heacock et al., 1984). To examine whether Derlin-1 and Derlin-2 regulate the transcription of cholesterol biosynthesis-related genes, total RNAs harvested from the cerebella of *Derl1*^*NesCre*^, *Derl2*^*NesCre*^, and control mice were analyzed using qPCR. Derlin-1 or Derlin-2 deficiency reduced the mRNA levels of many genes encoding components of the cholesterol biosynthetic pathway, *Hmgcs1*, *Hmgcr*, *Mvk*, *Fdft1*, *Cyp51*, and *Dhcr24* (Figure 5C). Moreover, total cholesterol was significantly reduced in the cerebella of *Derl1*^*NesCre*^ and *Derl2*^*NesCre*^ mice (Figures 5D and 5E).

Many cholesterol biosynthetic enzyme genes are induced by the transcription factor SREBP-2, which resides in the ER until translocation to the nucleus, suggesting that Derlins in the ER may normally serve to enhance cholesterol production by promoting nuclear SREBP-2 activity. To address this question directly, we compared expression of ER (precursor) and nuclear (active) forms of SREBP-2 in cerebellar membrane and nuclear extracts from knockout and control mice. To test the feasibility of this approach, we first examined expression in extracts from human hepatoma HepG2 cells, a line demonstrating robust SREBP-2 activity, under excess cholesterol and cholesterol depletion. The 125 kDa band in the membrane fraction and the 68 kDa band in the nuclear fraction from HepG2 cell extracts were also detected in cerebellar extract from wild-type C57BL/6 mouse (Figure S6A). The amount of precursor (p)SREBP-2 was significantly elevated in the cerebellar membrane fraction from *Derl1*^*NesCre*^ and *Derl2*^*NesCre*^ mice (Figures 5F, 5G, 5I, and 5J), whereas nuclear (n)SREBP-2 was significantly reduced (Figures 5F, 5H, 5I, and 5K). The ER-resident transcription factor SREBP-1 is also cleaved by the Golgi-resident site-1 and site-2 proteases and translocates to the nucleus, resulting in the activation of fatty acid synthesis and lipoprotein metabolism. However, lipoprotein and fatty acid metabolism-related genes were not enriched among the Derlin-1 downstream targets (Figures S6B–S6D) and the significant reduction of mRNA levels of SREBP-1-mediated genes *Acaca* and *Fasn* was not observed in the cerebellum of *Derl1*^*NesCre*^ mice compared with *Derl1*^*f/f*^ mice (Figure S6E). These results suggest that indeed Derlin-1 and Derlin-2 are required for the nuclear translocation and activation of SREBP-2 (at least in the cerebellum).

### Requirement of SREBP-2-mediated cholesterol biosynthesis for neurite outgrowth

**Figure 6:**
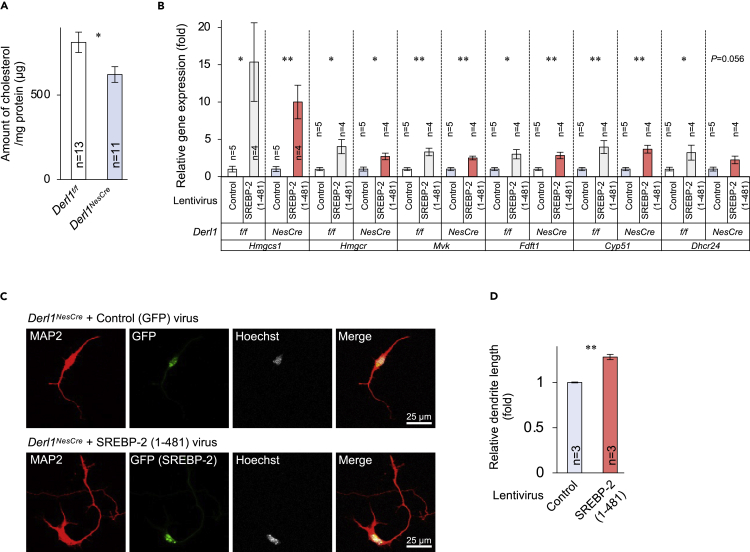
Rescue of shortened neurite outgrowth of Derlin-1-deficient cortical neuron by exogenously expressed active form of SREBP-2 (A) Quantification of the amount of cholesterol per milligram protein in 6 DIV primary cultured cortical neurons derived from *Derl1*^*f/f*^ and *Derl1*^*NesCre*^ embryos. (B–D) Rescue of shortened neurite outgrowth of Derlin-1-deficient neuron by active form of SREBP-2. Gene expression levels of cholesterol biosynthesis-related genes in neurons were estimated by qPCR and normalized to that of *S18* (B). Neurons were stained with anti-MAP2 and GFP antibodies (C). Dendritic length of MAP2-positive neuron and MAP2- and GFP-double-positive neuron was quantified using ImageJ software (D). More than 80 neurons were measured in each culture dish and averaged to obtain n = 1. Bar graphs are presented as mean ± SEM. ∗P < 0.05 and ∗∗P < 0.01 by Student's t test. n indicates the number of unrelated culture dishes. See also Figure S7, Table S3.

*Derl1*^*NesCre*^ embryo-derived cultured primary cortical neurons also exhibited reduced mRNA levels of several cholesterol biosynthesis-related genes (Figure S7A) and significantly lower cholesterol per milligram protein compared with *Derl1*^*f/f*^ mouse-derived neurons (Figure 6A). Taken together, these results suggest that Derlin-1 contributes to the maintenance of brain cholesterol biosynthesis. To investigate whether cholesterol biosynthesis would be required for neurite outgrowth, we treated the primary cultured cortical neurons with lovastatin, a strong cholesterol biosynthesis inhibitor that blocks HMG-CoA reductase. The treatment with lovastatin significantly increased cholesterol biosynthetic genes, suggesting that cholesterol was depleted in primary cortical neurons (Figure S7B). Lovastatin-treated neurons exhibited shortened neurite outgrowth (Figures S7C and S7D). To investigate whether SREBP-2 activation is sufficient to restore normal neurite outgrowth, primary cultured *Derl1*^*NesCre*^ neurons were transfected with lentivirus-encoded control Venus (GFP variant) or Venus-tagged human SREBP-2 (1–481), which activates the cholesterol biosynthetic pathway (Figure S7E), and expression levels of cholesterol biosynthetic genes and neurite length were compared. Consistent with impaired cholesterol synthesis under Derlin deficiency contributing to reduced neurite outgrowth, exogenously expressed SREBP-2 (1–481) significantly restored the reduced expression levels of biosynthetic genes (*Hmgcs1*, *Hmgcr*, *Mvk*, *Fdft1*, and *Cyp51*) in *Derl1*^*NesCre*^ neurons (Figure 6B) and increased neurite length (Figures 6C and 6D). Although there is a possibility that the overexpression of Derlins induces the neurite outgrowth independently of cholesterol biosynthesis, exogenously expressed Derlin-1 (Figure S7F) or Derlin-2 (Figure S7G) had no effect on the neurite outgrowth of wild-type cortical neurons in which cholesterol biosynthesis is not inhibited (Figures S7H and S7I). Collectively, our findings indicate that inhibition of SREBP-2-mediated cholesterol biosynthesis by deletion of *Derl1* or *Derl2* reduces neurite outgrowth and disrupts normal brain development, whereas we cannot exclude the possibility that the altered ERAD function by deletion of Derlins may contribute to shorter neurites to some extent. This abnormal development was particularly pronounced in the cerebellum and striatum, resulting in motor dysfunction.

## Discussion

In this work, we provide evidence that Derlin-1 and Derlin-2 have an unexpected function required for normal postnatal brain development and function. Both Derlin-1- and Derlin-2-deficient mice exhibited widespread postnatal brain atrophy, which was particularly severe in the cerebellum and striatum. Neurons in the cerebellum and striatum also exhibited reduced neurite outgrowth, and consistent with neural circuit dysfunction in these regions, both mouse lines demonstrated impaired motor performance. We speculate that these motor deficits are due to insufficient processing capacity by motor control circuits comprising these maldeveloped striatal and cerebellar neurons. An alternative explanation is that Derlin perturbs the UPR, which has also been reported to induce severe brain maldevelopment (Alimov et al., 2013; Passemard et al., 2019). However, we suggest that an ER stress response triggered by impaired ER quality control does not contribute to the observed postnatal brain atrophy in Derlin-deficient mice because the treatment of primary Derlin-1-deficient neurons with 4-PBA did not mitigate the shortened neurite outgrowth (Figure 4F).

In addition, we unexpectedly found that Derlin-1 or Derlin-2 deficiency resulted in reduced cholesterol biosynthesis within the brain. Cholesterol biosynthesis is necessary for glial cell proliferation, neurite outgrowth, microtubule stability, synapse formation, and myelination (Zhang and Liu, 2015); hence, a sufficient cholesterol supply to the brain must be maintained throughout development as these processes are important for adaptive plasticity and/or reparative functions. Indeed, impairment of cholesterol biosynthesis at any embryonic or perinatal stage of development results in CNS dysfunction (Cunningham et al., 2015; Driver et al., 2016; Fan et al., 2002; Goritz et al., 2005; Suzuki et al., 2007). Cellular cholesterol is tightly regulated by SREBP-2 (Brown and Goldstein, 1997; Luo et al., 2020), and we demonstrate that Derlin-1 or Derlin-2 is required for the activation of SREBP-2 and downstream target genes *in vivo*. Since Derlin deficiency alters the expression of other ERAD components (Figure 4C), altered ERAD function might contribute to the activation of SREBP-2 pathway. Moreover, an exogenously expressed active form of SREBP-2 mitigated the reduced dendritic length of primary cultured neurons from Derlin-1-deficient mice. Collectively, these findings suggest that SREBP-2-mediated cholesterol biosynthesis in neurons is regulated by Derlin-1 and Derlin-2 and that the maintenance of cholesterol biosynthesis may be critical for normal neurite outgrowth and postnatal brain development. Moreover, inhibition of SREBP-2 expression in cultured hippocampal neurons also induced synapse malfunction (Suzuki et al., 2010). However, SREBP-2 is expressed not only in neurons but also in astrocytes, microglial cells, and oligodendrocytes, and several groups have reported essential contributions of non-neuronal cells to total brain cholesterol biosynthesis. For example, around 80% of brain cholesterol is synthesized in oligodendrocytes that form myelin (Saher et al., 2011). In addition, maintenance of total neuronal cholesterol is dependent on a supply from astrocytes, and astrocyte-specific deletion of SREBP-2 in mice induced microcephaly and motor defects (Ferris et al., 2017). However, we found that neuron-specific Derlin-1 deletion alone induced brain atrophy (Figures 3G, S4B, and S4C) and motor dysfunction (Figures 3H and 3I). Similarly, siRNA-mediated knockdown of SREBP in *Drosophila* neurons, but not local glial cells, reduced neurite length (Ziegler et al., 2017), indicating that this process can be mediated by cell-autonomous cholesterol synthesis. Although we cannot eliminate the contribution of cholesterol from non-neural cells, since there are only neurons without other sustaining cells in our culture assay of primary cortical neurons (Figures 2H, 2I, 6A–6D, and S7B–S7D), the current observations strongly suggest that neuronal cholesterol biosynthesis regulated by Derlins is indispensable for normal neurite outgrowth and postnatal brain development.

In conclusion, our findings demonstrate an unexpected function of Derlins in brain development via regulation of cholesterol biosynthesis. Although further investigation is necessary to clarify the precise mechanisms by which Derlin-1 and Derlin-2 regulate SREBP-2 activation, we propose that Derlins may be a therapeutic target to ameliorate or delay the progression of neurodegenerative diseases.

### Limitations of the study

An important question is how Derlin-1 and Derlin-2 regulate the SREBP-2 pathway. When excess cholesterol accumulates in the ER membrane, Insigs prevent recruitment of the Scap/SREBP-2 complex to COPII-coated vesicles and, thereby, halt SREBP-2 transport to the Golgi apparatus. Insig-1 is ubiquitinated by gp78 and degraded by the ERAD pathway during cholesterol depletion, whereas the sterol-induced binding of Insig-1 to Scap prevents degradation (Gong et al., 2006; Tsai et al., 2012). Because Derlin-1 interacts and coordinates with gp78 in the ERAD complex (Bernardi et al., 2010; Kadowaki et al., 2018), Derlin deficiency may stabilize Insig-1 and anchor Scap/SREBP-2 to the ER membrane. Indeed, SREBP-2 immunoreactivity was elevated in the membrane fraction but reduced in the nuclear fraction of cerebellar lysates from Derlin-deficient mice (Figures 5F and 5I). Since the significant down-regulation of the SREBP-1 pathway was not observed in the cerebella of *Derl1*^*NesCre*^ mice (Figures S6B–S6E), there might be a specific Derlin-mediated regulatory mechanism for SREBP-2 activation. Further studies are needed to determine the precise molecular mechanisms by which Derlins regulate SREBP-2 nuclear translocation and brain cholesterol biosynthesis.

## STAR★Methods

### Key resources table

**Table:** 

REAGENT or RESOUCE	SOURCE	IDENTIFIER
**Antibodies**		

Rabbit-anti-Derlin-1	(Nishitoh et al., 2008)	N/A
Rabbit-anti-Derlin-2	MBL International	PM019; RRID: AB_593007
Mouse-anti-Actin	Sigma-Aldrich	A4700; RRID: AB_476730
Rabbit-anti-SREBP2	Abcam	ab30682; RRID: AB_779079
Mouse-anti-Lamin B1	Thermo Fisher Scientific	33-2000; RRID: AB_2533106
Rabbit-anti-Calnexin	Abcam	ab22595; RRID: AB_2069006
Mouse-anti-NeuN	Millipore	MAB377; RRID: AB_2298772
Mouse-anti-Calbindin-D-28K	Sigma-Aldrich	C9848; RRID: AB_476894
Chicken-anti-Glial Fibrillary Acidic Protein	Millipore	AB5541; RRID: AB_177521
Mouse-anti-S-100 (β-Subunit)	Sigma-Aldrich	s2532; RRID: AB_477499
Rabbit-anti-Iba1	Wako	019-19741; RRID: AB_839504
Rabbit-anti-Tubulin β-3	Covance	PRB-435P; RRID: AB_2564645
Guinea pig-anti-MAP2	Synaptic System	188 004; RRID: AB_2138181
Mouse-anti-MAP2	Sigma-Aldrich	M4403; RRID: AB_477193
Mouse-anti-GFP	MBL International	M048-3; RRID: AB_591823
Rabbit-anti-SEL1L	Abcam	ab78298; RRID: AB_2285813
Rabbit-anti-HRD1	Sigma-Aldrich	H7915; RRID: AB_1840939
Rabbit-anti-P-eIF2α	Invitrogen	44-728G; RRID: AB_1500038
Mouse-anti-BiP	MBL International	M181-3; RRID: AB_10693914
Rabbit-anti-IRE1α	Cell signaling	3294; RRID: AB_823545
Rabbit-anti-PERK	Cell signaling	3192; RRID: AB_2095847
Rabbit-anti-OS9	Abcam	ab109510; RRID: AB_2864354
Rabbit-anti-gp78	Proteintech	16675-1-AP; RRID: AB_2226463
Anti-rabbit IgG, HRP-linked antibody	Cell signaling Technology	7074; RRID: AB_2099233
Anti-Mouse IgG, HRP-linked antibody	GE Healthcare	NA931; RRID: AB_772210
CF®555, Donkey Anti-Mouse IgG (H+L), Highly Cross-Adsorbed	Biotium	20037; RRID: AB_10559035
CF®555, Donkey Anti-Rabbit IgG (H+L), Highly Cross-Adsorbed	Biotium	20038; RRID: AB_10558011
CF®647, Donkey Anti-Rabbit IgG (H+L), Highly Cross-Adsorbed	Biotium	20047; RRID: AB_10559808
CF®488A, Donkey Anti-Chicken IgY (H+L), Highly Cross-Adsorbed	Biotium	20166; RRID: AB_10854387
CF®488A, Donkey Anti-Mouse IgG (H+L), Highly Cross-Adsorbed	Biotium	20014; RRID: AB_10561327
Cy™5 AffiniPure Donkey Anti-Guinea Pig IgG (H+L)	Jackson ImmunoResearch Labs	706-175-148; RRID: AB_2340462

**Bacterial and Virus Strains**		

LV-pRRL-Venus-HA	Nishitoh et al. (2008)	N/A
LV-pRRL-Venus-hSREBP2(1‒481)-HA	This paper	N/A
LV-pRRL-mDerlin-1-HA	This paper	N/A
LV-pRRL-mDerlin-2-HA	This paper	N/A

**Biological Samples**		

Mouse brain tissue	Strains listed in this table	N/A

**Chemicals, Peptides, and Recombinant Proteins**		

Tissue Tek	Sakura Finetek	Cat# 4583
Immu-Mount	Thermo Scientific	Cat# 9990402
Dulbecco’s modified Eagle’s medium	Nacalai Tesque	Cat# 08459-64
Minimal essential medium (MEM)	Nacalai Tesque	Cat# 21443-15
Neurobasal	Gibco	Cat# 21103-049
MEMα	Gibco	Cat# 12571-063
Penicillin-streptomycin solution	Nacalai Tesque	Cat# 09367-34
Bisbenzimide H33258 Fluorochrome Trihydrochloride Solution	Nacalai Tesque	Cat# 19173-41
Poly-L-lysine hydrobromide	Sigma-Aldrich	Cat# P2636
Papain from papaya latex	Sigma-Aldrich	Cat# P3125
DNase I	Sigma-Aldrich	Cat# D4527
B-27 supplement	Gibco	Cat# 17504-044
GlutaMAX supplement	Gibco	Cat# 35050-061
Cytosine β-D-arabinofuranoside crystalline	Sigma-Aldrich	Cat# C1768
4-phenylbutyric acid	MERCK	Cat# 8.20986.0025
Bovine lipoprotein deficient serum	Alpha Diagnostic Int	Cat# LDLD46-S
Protease Inhibitor Cocktail for Use with Mammalian Cell and Tissue Extracts	Nacalai Tesque	Cat# 25955-11
ALLN	Calbiochem	Cat# 208719
Leupeptin	Nacalai Tesque	Cat# 43449-62
Lovastatin	AdipoGen	Cat# AG-CN2-0051
25-hydroxycholesterol	Sigma-Aldrich	Cat# H1015
Synthetic cholesterol	Sigma-Aldrich	Cat# S5442
Polyethylenimine (PEI)-Max	Polysciences	Cat# 24765-1
Lipofectamine RNAiMAX reagent	Invitrogen	Cat# 13778150

**Critical Commercial Assays**		

FD Rapid GolgiStain Kit	FD NeuroTechnologies	Cat# PK401
RNAiso Plus	Takara Bio	Cat# 9109
Rneasy Plus Mini Kit	QIAGEN	Cat# 74104
NucleoSpin RNA kit	Takara Bio	Cat# 740955
RevaTra Ace qPCR RT Master Mix with gDNA Remover	TOYOBO	Cat# FSQ-301
SYBR Green PCR Master Mix	Kapa Biosystems	Cat# KK4602
SurePrint G3 Mouse GE Ver2 platform	Agilent Technologies	Cat# G4852A, G4852B
Colorimetric total cholesterol assay kit according to manufacturer instructions	Cell Biolabs	Cat# STA-384

**Deposited Data**		

Raw and analyzed data	This paper	GEO: GSE155425 (https://www.ncbi.nln.nih.gov/geo/querq/acc.cgi?acc=GSE155425) GEO: GSE171796 (https://www.ncbi.nlm.nih.gov/geo/query/acc.cgi?acc=GSE171796)

**Experimental Models: Cell Lines**		

Human: HEK293T cells	ATCC	Cat# CRL-3216
Human: HepG2 cells	ATCC	N/A

**Experimental Models: Organisms/Strains**		

Mouse: C57BL/6 *Derl1*^*f/f*^	This paper	N/A
Mouse: C57BL/6 *Derl2*^*f/f*^	(Dougan et al., 2011)	N/A
Mouse: C57BL/6 *Tg(Nes-Cre)1Kag*	(Isaka et al., 1999)	N/A
Mouse: C57BL/6 *TgN(a-CaMKII-nlCre)/10*	(Karpati et al., 2019)	RBRC00153

**Oligonucleotides**		

Stealth RNAi™ siRNA SREBF2-HSS110189, target sequence: 5’-GAGGCAGGCUUUGAAGACGAAGCUA-3’	Invitrogen	Cat# 1299001
Stealth RNAi™ siRNA Negative Control Med GC Duplex #2	Invitrogen	Cat# 12935112
Primers for quantitative real-time PCR	See Table S3	N/A

**Recombinant DNA**		

pRRL-Venus-HA	(Nishitoh et al., 2008)	N/A
pRRL-Venus-hSREBP2(1-481)-HA	This paper	N/A
pRRL-mDerlin1-HA	This paper	N/A
pRRL-mDerlin2-HA	This paper	N/A
pMD2.G	Addgene	Cat# 12259
psPAX2	Addgene	Cat# 12260
pcDNA3.0	Invitrogen	N/A

**Software and algorithms**		

ImageJ	(Schneider et al., 2012)	https://imagej.nih.gov/ij/
Fiji	(Schindelin et al., 2012)	https://imagej.net/Fiji
Adobe Photoshop Elements 14	Adobe	N/A
ImageQuant TL Ver.8.1	GE Healthcare	N/A
GeneSpring software version 13.0, 14.9, 14.9.1	Agilent Technologies	N/A
DAVID 6.8	(Huang da et al., 2009)	http://david.ncifcrf.gov/
GSEA v4.0.3	(Mootha et al., 2003; Subramanian et al., 2005)	https://www.gsea-msigdb.org/gsea/index.jsp
MeV	Center for Cancer Computational Biology at Dana-Farber Cancer Institute	http://mev.tm4.org/
Mouse Genome Informatics	The Jackson Laboratory	http://www.informatics.jax.org/
EZR software version1.30	(Kanda, 2013)	http://www.jichi.ac.jp/saitama-sct/SaitamaHP.files/statmed.html

**Other**		

Micro Smash	TOMY	Cat# MS-100
Freezing microtome	Leica Microsystems	Cat# CM3050S
Confocal laser microscope	Leica Microsystems	Cat# TSC-SP8
Fluorescence microscope	Keyence	Cat# BZ-9000
ROTA-ROD FOR MICE	UGO Basile	Cat# 47600
ROTA-ROD TREADMILL FOR MICE	Muromachi	Cat# MK-610A
Balanced beam test	O’Hara	N/A

### Resource availability

#### Lead contact

Further information and requests for resources and reagents should be directed to and will be fulfilled by the Lead Contact, Hideki Nishitoh (nishitoh@med.miyazaki-u.ac.jp)

#### Materials availability

All unique reagents (plasmids, antibodies, and *Derl1*^*f/f*^ mice) generated in this study are available from the Lead Contact. *Derl2*^*f/f*^ mice are available from Hidde L. Ploegh. *C57BL/6-TgN(a-CaMKII-nlCre)/10* mice are available from Katsuhiko Mikoshiba. *Tg(Nes-Cre)1Kag* mice are available from Ryoichiro Kageyama (Kyoto University).

#### Data and code availability

DNA microarray data generated in this study are deposited with the NCBI Gene Expression Omnibus archive as series GSE155425 and GSE171796 and are publicly available as of the date of publication. This paper does not report original code. Any additional information required to reanalyze the data reported in this paper is available from the lead contact upon request.

### Experimental model and subject details

#### Animals

Wild-type C57BL/6 mice were raised under specific pathogen-free conditions and housed under a 12-h/12-h light/dark cycle with free access to food and water. *Derl1*^*f/f*^ mice were generated by conventional gene targeting (Figure S1A) as previously described, while mice expressing Cre recombinase driven by the *nestin* promoter [*Tg(Nes-Cre)1Kag* mice] were obtained from Dr. Ryoichiro Kageyama (Kyoto University) (Isaka et al., 1999). Mice expressing Cre recombinase driven by the *CaMKIIα* promoter [*C57BL/6-TgN(a-CaMKII-nlCre)/10*] have been described (Karpati et al., 2019). Mice expressing Cre recombinase were intercrossed with *Derl1*^*f/f*^ mice and *Derl2*^*f/f*^ mice to generate *Derl1*^*NesCre*^, *Derl2*^*NesCre*^, and *Derl1*^*CaMKIIαCre*^ mice. Both male and female mice were used, as mice up to 5 weeks old are sexually immature and do not affect the results. After 6 weeks of age, male mice were mainly used to avoid the influences of the female sex cycle, including behavioral analysis. All mice experiments were approved by the Animal Research Committee of the University of Miyazaki, National Institute for Physiological Sciences, and University of Toyama and performed in accordance with the institutional guidelines. The experiments were performed in accordance with the institutional guidelines. All efforts were made to minimize animal suffering and to reduce the number of animals used.

#### Primary cultures of cortical neurons

Cortical neurons were isolated from E17 wild-type, *Derl1*^*f/f*^ and *Derl1*^*NesCre*^ mice (female and male). In brief, cerebral cortices were dissociated with papain (Sigma-Aldrich; P3125) at 37°C for 20 min, and triturated in the presence of DNase I (Sigma-Aldrich; D4527) and 10% fetal bovine serum (FBS). Dissociated cells were plated on culture dishes pre-coated with poly-L-lysine (Sigma-Aldrich; P2636) in minimal essential medium (MEM)α (Gibco; 12571-063) supplemented with 5% FBS and 0.6% glucose. After allowing neurons to adhere for 3 to 4 h, the plating medium was replaced with Neurobasal medium (Gibco; 21103-049) supplemented with 20 μL/mL B27 (Gibco; 17504-044) and 0.5 mM GlutaMAX (Gibco; 35050-061). To eliminate non-neuronal cells, 5 μM cytosine β-D-arabinofuranoside crystalline (Sigma-Aldrich; C1768) was added. 4-phenylbutyric acid (4-PBA) (1 mM) (MERCK; 8.20986.0025) and lovastatin (1 μM) (AdipoGen; AG-CN2-0051) were treated at the first medium replace timing. A half-volume of medium was replaced every three days. The cells were maintained in a 5% CO_2_ atmosphere at 37°C.

### Method details

#### Cell culture

Human hepatoma HepG2 cells were cultured in MEM (Nacalai Tesque; 21443-15) supplemented with 10% FBS and penicillin–streptomycin solution (Nacalai Tesque; 09367-34). Human embryonic kidney (HEK) 293T cells were cultured in Dulbecco’s modified Eagle’s medium (Nacalai Tesque; 08459-64) supplemented with 10% FBS and penicillin–streptomycin solution. All cells were maintained under a 5% CO_2_ atmosphere at 37°C.

#### Tissue preparation for biochemical analysis

Mice were sacrificed by cervical dislocation and brains rapidly dissected for immunoblotting (IB), qPCR, and DNA microarray analysis. Each brain region was frozen immediately on dry ice and stored at −80°C.

#### Tissue preparation for immunofluorescence

Mice were deeply anesthetized by intraperitoneal injection of a 4 mg/kg midazolam/0.3 mg/kg medetomidine/5 mg/kg butorphanol mixture, and transcardially perfused with phosphate buffered saline (PBS) followed by 4% paraformaldehyde (PFA) in PBS. Brains were dissected and post-fixed overnight in the same fixative at 4°C. Fixed brains were incubated in 15% sucrose solution at 4°C overnight followed by incubation in 30% sucrose solution at 4°C overnight. The size of fixed brains was measured before embedding. Brains were then cut into two pieces along the midline, and each half was embedded in optimal cutting temperature compound (Tissue Tek; Sakura Finetek; 4583) and stored at −80°C. Embedded frozen brains were serially sectioned in the coronal plane at 40-μm thickness using a freezing microtome (Leica Microsystems; CM3050S), and every sixth section was sequentially transferred to 6-well plates in PBS for subsequent immunohistochemical staining (below).

#### Immunohistochemistry

The brain sections were washed with PBS and incubated in blocking solution (PBS containing 3% FBS and 0.1% Triton X-100) for 1 h at room temperature (RT) followed by overnight incubation at 4°C with the indicated primary antibody diluted in blocking solution. Sections were washed thrice with PBS and incubated for 2 h at RT with secondary antibody diluted in blocking solution. After a final wash with PBS, the sections were mounted on glass slides with Immu-Mount (Thermo Scientific; 9990402). Immunofluorescence images were obtained using a fluorescence microscope (Keyence; BZ-9000) or confocal laser microscope (Leica Microsystems; TSC-SP8) and processed using Adobe Photoshop Elements (Adobe). Nuclei were counterstained using bisbenzimide H33258 fluorochrome trihydrochloride solution (Hoechst; 1:500; Nacalai Tesque, 19173-41). Antibodies are listed in the key resources table.

#### Golgi staining

Coronal half brain sections from 30-week-old *Derl1*^*NesCre*^ and *Derl2*^*NesCre*^ mice and their respective controls were stained using the FD Rapid GolgiStain Kit (FD NeuroTechnologies; PK401) following the manufacturer’s recommendations and then cut into 150-μm thick coronal sections on a cryostat (Leica Microsystems). All sections were visualized by confocal laser microscopy (Leica Microsystems). Ten cerebellar Purkinje cells and 10 striatal MSNs were analyzed from 3 unrelated animals per genotype. For MSNs, dendrite length, branch number, and number of branches at given distances from the soma were measured using the Sholl analysis plug-in of Fiji software (National Institutes of Health) after neuronal reconstruction with the plug-in Simple Neurite Tracer. The dendritic tree size of Purkinje cells was measured using ImageJ software (National Institutes of Health), and the number of branches was counted manually.

#### Volumetric analysis and cell counting

Volumetric analyses were conducted using every sixth 40-μm coronal half brain section stained with NeuN. The areas of each brain region were measured using ImageJ and volume (V) calculated as V = ΣA × i × d according to the Cavalieri’s principle, where A is the sum of target areas in each section, i is the interval between the sections, and d is the section thickness. Fold changes between *Derl1*^*NesCre*^ or *Derl2*^*NesCre*^ mice and respective controls were calculated as measures of regional brain atrophy. Marker-positive cell numbers in the cerebellum and striatum were calculated using every sequential hemisphere section. The number of Purkinje cell somata per 200 μm of the Purkinje cell layer was manually counted at 12 sites, and the average was compared among genotypes. The numbers of marker-positive cells were also manually counted within twelve 150 × 150 μm areas of the cerebellar molecular layer and twelve 200 × 200 μm areas of the striatum. These cell numbers are reported per mm^2^.

#### Immunoblotting

Whole cell lysates were prepared by homogenizing brain and other tissues for 60 s in lysis buffer (20 mM Tris-HCl pH 7.5, 150 mM NaCl, 5 mM EGTA, and 1% Triton X-100) supplemented with 5 μg/mL leupeptine (Nacalai Tesque; 43449-62) on ice using a Micro Smash (TOMY; MS-100) (4,500 rpm 4°C). Cellular nuclear and membrane fractions were isolated as described previously with minor modifications (Sakai et al., 1996). Briefly, brain tissues were homogenized in 0.5 mL of buffer A (10 mM HEPES at pH 7.6, 10 mM KCl, 1.5 mM MgCl_2_, 1.0 mM EDTA, 1.0 mM EGTA) containing protease inhibitor cocktail (Nacalai Tesque; 25955-11) and ALLN (Calbiochem; 208719). Cells were allowed to swell in the homogenate at 4°C for 30 min and then passed through a 23-gauge needle 30 times. The obtained lysate was centrifuged at 1,000 ×*g* for 7 min at 4°C. The supernatant from this 1,000 ×*g* centrifugation was used to prepare the membrane fraction. First, the supernatant was centrifuged at 100,000 ×*g* for 30 min at 4°C, followed by resuspension of the pellet in 0.25 mL of lysis buffer containing 5 μg/mL leupeptine. The new suspension was centrifuged at 20,400 ×*g* at 4°C for 15 min, and the supernatant was used as the membrane fraction. Alternatively, the pellet obtained from the 1,000 ×*g* centrifugation above was used to isolate the nuclear fraction. The pellets were resuspended in 0.25 mL of buffer B (20 mM HEPES at pH 7.6, 2.5% glycerol, 0.42 M NaCl, 1.5 mM MgCl_2_, 1.0 mM EDTA, and 1.0 mM EGTA) containing protease inhibitor cocktail and ALLN. The suspension was centrifuged at 20,400 ×*g* at 4°C for 15 min. The supernatant from this spin was used as the nuclear fraction.

Whole cell lysates, nuclear fractions, and the membrane fractions were resolved by sodium dodecyl sulfate-polyacrylamide gel electrophoresis (SDS-PAGE) and blotted onto polyvinylidene fluoride (PVDF) membranes. After blocking with 5% skim milk in TBS-T (50 mM Tris-HCl pH 8.0, 150 mM NaCl and 0.05% Tween-20), the membranes were probed with the indicated antibodies and immunolabeling detected with an enhanced chemiluminescence (ECL) system. Antibodies are listed in the key resources table. Band intensity was measured by ImageQuant TL (GE Healthcare).

#### Evaluating SREBP-2 processing in cultured cells

HepG2 cells were plated in MEM supplemented with 10% FBS and penicillin–streptomycin solution (Nacalai Tesque), and transfected with a small interfering RNA (siRNA) targeting SREBF2 (SREBF2-HSS110189 Stealth siRNA; Invitrogen; 1299001) or control siRNA (Negative Control Medium GC Duplex; Invitrogen; 12935112) using Lipofectamine RNAiMAX reagent (Invitrogen; 13778150). After two days (defined as 2 DIV), each culture was washed twice with PBS and switched to medium for inducing SREBP-2 activation (minus sterols) or suppressing SREBP-2 activation (plus sterols) as described previously with minor modifications (Hua et al., 1995; Sakai et al., 1996). Medium for inducing SREBP-2 activation included 5% bovine lipoprotein-deficient serum (LPDS) (Alpha Diagnostic Int; LDLD46-S) and 10 μM lovastatin (AdipoGen; AG-CN2-0051) in MEM (Nacalai Tesque), while the medium for suppressing SREBP-2 activation included 5% LPDS, 1 μg/mL 25-hydroxycholesterol (Sigma-Aldrich; H1015), and 10 μg/mL cholesterol (Sigma-Aldrich; S5442) in MEM. After incubation for 16–20 h in induction or suppression medium (3 DIV), 25 μg/ml ALLN (Calbiochem) was added to each dish and the cells were harvested 2–4 h later.

#### Lentivirus production and infection

Lentiviruses were produced by co-transfecting HEK293T cells with the lentivirus constructs pRRL-Venus-HA, pRRL-Venus-hSREBP-2(1‒481)-HA, pRRL-mDerlin-1-HA or pRRL-mDerlin-2-HA and lentivirus packaging vector constructs pMD2.G (Addgene; 12259) and psPAX2 (Addgene; 12260)] using Polyethylenimine (PEI)-Max (Polysciences; 24765-1). The culture medium was changed at 16–24 h after transfection. The supernatants were collected at 24 and 48 h after medium change, and virus was introduced into cortical neurons by adding these supernatants to the culture at the first medium change.

#### Immunocytochemistry

Primary cultured cortical neurons were fixed with 4% PFA in PBS for 20 min at the indicated times (DIV), washed thrice in PBS, permeabilized and blocked with blocking solution (PBS containing 3% FBS and 0.1% Triton X-100) for 30 min at RT, and incubated for 1.5 h at RT with the indicated primary antibody diluted in blocking solution. Cells were then washed thrice with PBS and incubated for 1.5 h at RT with the secondary antibody diluted in blocking solution. After a final wash with PBS, cells were mounted on glass slides with Immu-Mount (Thermo Scientific). Nuclei were counterstained using Hoechst (1:500; Nacalai Tesque). Immunofluorescence images were obtained using a confocal laser microscope (Leica Microsystems) and processed using Adobe Photoshop Elements. The antibodies are listed in key resources table.

#### Morphological analysis of cultured cortical neurons

For analysis of dendrite development *in vitro*, cortical neurons were immunostained with antibodies against MAP2 at 3 DIV (Figures 4E, 6C, and S7E) and 6 DIV (Figure 2H). Dendrites were defined as MAP2-positive neurites. Dendritic length was quantified using ImageJ.

#### Quantitative real-time PCR analysis

Total RNA was isolated from the cerebellum at P28 or cultured cortical neurons at 3 DIV using RNAiso Plus (Takara Bio; 9109) or the RNeasy Plus Mini Kit (QIAGEN; 74104) and reverse transcribed using RevaTra Ace qPCR RT Master Mix with gDNA Remover (TOYOBO; FSQ-301). Quantitative (q)PCR was performed using SYBR Green PCR Master Mix (Kapa Biosystems; KK4602) and a StepOnePlus Real-Time PCR System (Applied Biosystems). Expression levels were normalized to expression of S18 mRNA. The primer sequences are shown in Table S3.

#### DNA microarray analysis

Total RNA was extracted from the cerebellum at P28 using a NucleoSpin RNA kit (Takara Bio; 740955) according to the manufacturer’s instructions. A total of 150 ng total RNA from each sample was amplified and Cy3-labeled. Next, 600 ng Cy3-labeled cRNA was fragmented, hybridized onto the SurePrint G3 Mouse GE Ver2 platform (Agilent Technologies; G4852B and G4858A) and then incubated with rotation at 65°C for 17 h. Data were analyzed using GeneSpring software version 13.0 and 14.9 (Agilent Technologies) as previously described (Komatsu et al., 2013). In brief, the microarray data were normalized by quantile normalization, and baseline transformed the signal values to the median in all samples. Then, quality control and filtering steps were performed based on flags and expression levels. Mean signal intensities were measured in duplicate and averaged for identification of genes differentially expressed among mouse lines. A fold-change <0.67 was considered downregulation and a fold-change >1.5 as upregulation. Data from this microarray analysis have been submitted to the NCBI Gene Expression Omnibus archive as series GSE155425 and GSE171796. Functional enrichment analysis of differentially expressed genes was performed using DAVID online tools (version DAVID 6.8; http://david.ncifcrf.gov/). Heat map analysis was performed using MeV (multiple experimental viewer; http://mev.tm4.org/). On the heat map, red indicates higher expression and green lower expression. Gene set enrichment analysis (GSEA) was performed using GSEA v4.0.3 (https://www.gsea-msigdb.org/gsea/index.jsp). The Enrichment plot shows the distribution of genes in each set that are positively (red) and negatively (blue) correlated with Derlin-1 or Derlin-2 deficiency. The gene ontology (GO) terms for heat map analysis and GSEA were obtained from the Mouse Genome Informatics (MGI) GO project (http://www.informatics.jax.org/), which provides functional annotations for mouse gene products using Gene Ontology (http://www.informatics.jax.org/vocab/gene_ontology).

#### Cholesterol assay

Total cholesterol was measured from the cerebellum and primary cultured cortical neurons using a colorimetric assay kit according to the manufacturer’s instructions (Cell Biolabs; STA-384).

#### Rotarod test

*Derl1*^*f/f*^ and *Derl1*^*NesCre*^ mice were examined using an accelerating rotarod [ROTA-ROD FOR MICE (UGO Basile; 47600)], while *Derl2*^*f/f*^ and *Derl2*^*NesCre*^ mice were examined using the ROTA-ROD TREADMILL FOR MICE (Muromachi; MK-610A). In each trial, a mouse was placed on a rotating drum (3 cm diameter) and the time required to lose balance (as indicated by falling off or splaying out on the drum) was recorded. The speed of the rotarod was increased from 4 to 40 rpm over a 5 min period during the test. Each mouse performed 3 trials per day for a total of 6 trials over two days.

#### Beam-walking test

The balance beam apparatus (O’Hara) used for testing *Derl1*^*f/f*^, *Derl1*^*NesCre*^, and *Derl1*^*CaMKIIαCre*^ mice consisted of a 1-m cylindrical rod suspended horizontally 50 cm above the floor and connected to a safe platform, while the apparatus used for *Derl2*^*f/f*^ and *Derl2*^*NesCre*^ mice consisted of a 1-m long steel pipe suspended 30 cm above the floor and connected to a safe platform. In each test, a mouse was placed on the rod or pipe at the starting end, and the numbers of animals per genotype able to traverse to the safe platform as well as the time required was recorded. *Derl1*^*NesCre*^ and *Derl2*^*NesCre*^ mice and their respective control mice were tested on 3 trials while *Derl1*^*CaMKIIαCre*^ mice and controls underwent 1 trial.

### Quantification and statistical analysis

All data are presented as means ± standard error. Student’s *t*-test and Fisher’s exact test were performed to compare two group means. One-way ANOVA and repeated measures ANOVA followed by post hoc tests were used to compare three or more group means. All statistical analyses were performed by using EZR software version1.30 (Kanda, 2013). A P < 0.05 (two-tailed) was considered significant for all tests.

## Supplemental information

Document S1. Figures S1–S7

Table S1. Gene list whose expression level changed by more than 1.5-fold or less than 0.67-fold at postnatal day 28 in the cerebellum of Derlin-1-deficient mice, related to figure 4

Table S2. Gene list whose expression level changed by more than 1.5-fold or less than 0.67-fold at postnatal day 28 in the cerebellum of Derlin-2-deficient mice, related to figure 4

Table S3. Primers for quantitative real-time PCR, Related to key resources table
