# Supplementary material for: ERAD components Derlin-1 and Derlin-2 are essential for postnatal brain development and motor function
Source: iScience. 2021 Jun 19;24(7):102758. doi: 10.1016/j.isci.2021.102758 (PMC8324814; doi:10.1016/j.isci.2021.102758)
Supplement: Document S1. Figures S1–S7 [file mmc1.pdf]

iScience, Volume *24*

Supplemental information

ERAD components Derlin-1 and Derlin-2 are essential for postnatal brain development and motor function

Takashi Sugiyama, Naoya Murao, Hisae Kadowaki, Keizo Takao, Tsuyoshi Miyakawa, Yosuke Matsushita, Toyomasa Katagiri, Akira Futatsugi, Yohei Shinmyo, Hiroshi Kawasaki, Juro Sakai, Kazutaka Shiomi, Masamitsu Nakazato, Kohsuke Takeda, Katsuhiko Mikoshiba, Hidde L. Ploegh, Hidenori Ichijo, and Hideki Nishitoh

Figure S1

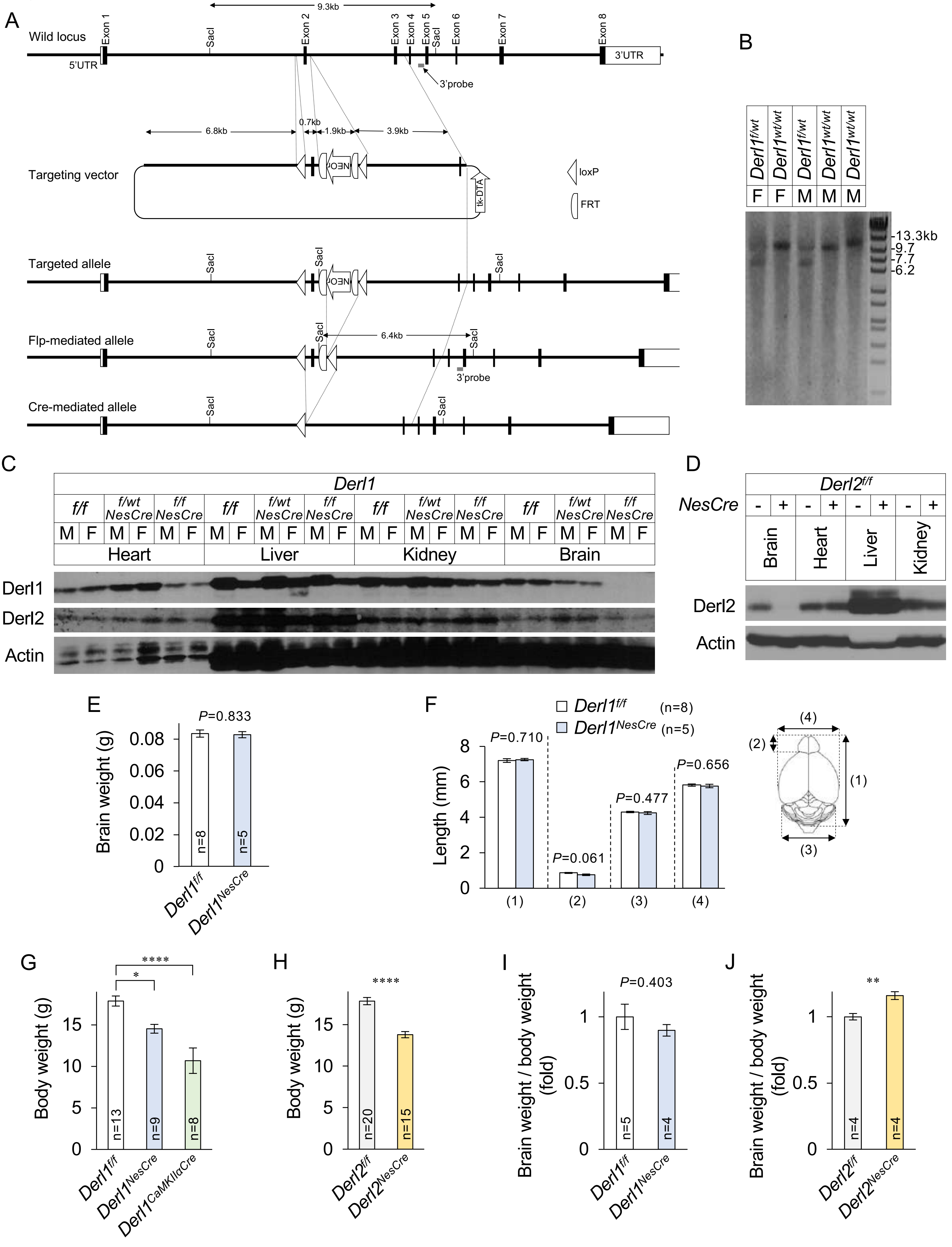
A
Wild locus
Exon 1
SacI
Exon 2
Exon 3
Exon 4
Exon 5
Exon 6
Exon 7
Exon 8
9.3kb
5'UTR
3'UTR
3'probe
Targeting vector
6.8kb
0.7kb
1.9kb
3.9kb
NEO
tk-DTA
loxP
FRT
Targeted allele
Flp-mediated allele
6.4kb
Cre-mediated allele
B
F
M
13.3kb
9.7
7.7
6.2
C
Derl1
f/f
f/wt NesCre
f/f NesCre
Heart
Liver
Kidney
Brain
Derl2
Actin
D
Derl2f/f
NesCre
E
Brain weight (g)
P=0.833
n=8
n=5
Derl1f/f
Derl1NesCre
F
(n=8)
(n=5)
Length (mm)
P=0.710
P=0.061
P=0.477
P=0.656
G
Body weight (g)
n=13
n=9
Derl1CaMKIIaCre
H
n=20
n=15
Derl2NesCre
I
Brain weight / body weight (fold)
P=0.403
n=4
J

Figure S1. Generation of CNS-specific Derlin-1- and Derlin-2-deficient mice, related to Figure 1

(A) Schematic diagram of the *Derl1* genomic locus, targeting construct, and the genomic locus after Cre-mediated deletion.

(B) Southern blotting of the SacI-digested *Derl1* genomic locus from *Derl1*$^{f/wt}$ and *Derl1*$^{wt/wt}$ mice. Correct targeting of the *Derlin-1* locus was indicated by the presence of a ~6.4 kb band on agarose gels. M, male; F, female.

(C and D) Expression of Derlin-1 and Derlin-2 in the heart, liver, kidney, and brain of *Derl1*$^{f/f}$, *Derl1*$^{f/wt:NesCre}$, and *Derl1*^{NesCre} mice (C) and *Derl2*$^{f/f}$ and *Derl2*^{NesCre} mice (D) aged 8 weeks. Whole tissue lysates from each organ were analyzed by immunoblotting (IB) with the indicated antibodies. *NesCre* (+), *Derl2*^{NesCre}; *NesCre* (−), *Derl2*$^{f/f}$. M, male; F, female.

(E and F) Comparison of *Derl1*$^{f/f}$ and *Derl1*^{NesCre} mouse brain weights (E) and lengths (F) at P0. Image of brain in *F* denotes the distances measured.

(G and H) The mean body weight of *Derl1*^{NesCre}, *Derl1*$^{CaMKII\alpha Cre}$, and *Derl2*^{NesCre} mice was less than that of control mice aged 4-5 weeks.

(I and J) The ratio of brain weight to body weight of *Derl1*^{NesCre} and *Derl2*^{NesCre} mice aged 4 weeks.

Bar graphs are presented as mean ± SEM. **P* < 0.05, ***P* < 0.01, and *****P* < 0.0001 by Student's *t*-test (E, F, and H–J) or one-way ANOVA (G, comparison of three groups). n indicates the number of animals.

Figure S2

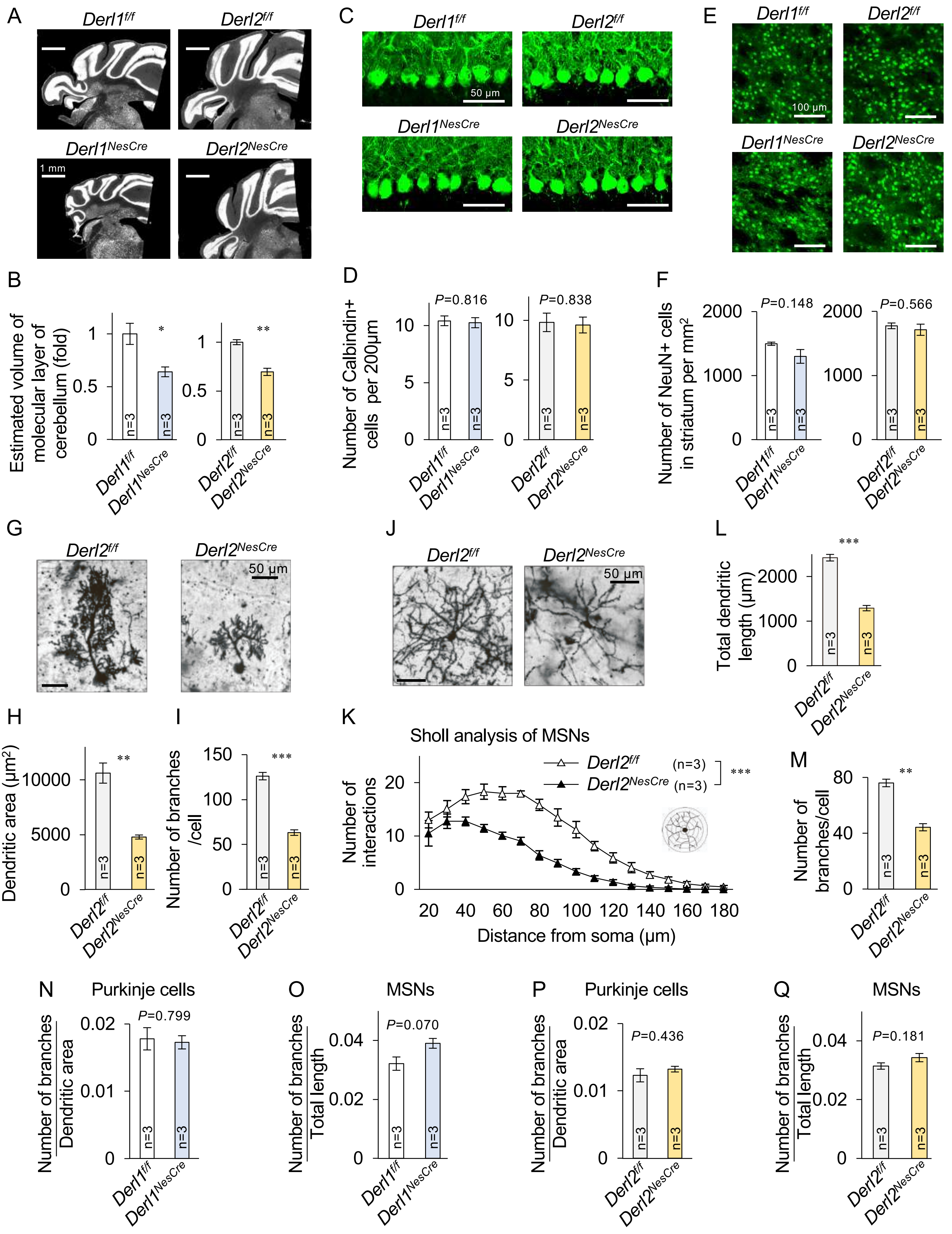

A
Derl1f/f
Derl2f/f
Derl1NesCre
Derl2NesCre
1 mm
B
Estimated volume of molecular layer of cerebellum (fold)
n=3
C
50 μm
D
Number of Calbindin+ cells per 200μm
P=0.816
P=0.838
E
100 μm
F
Number of NeuN+ cells in striatum per mm2
P=0.148
P=0.566
G
50 μm
H
Dendritic area (μm2)
I
Number of branches /cell
J
K
Sholl analysis of MSNs
Derl2f/f (n=3)
Derl2NesCre (n=3)
Number of interactions
Distance from soma (μm)
L
Total dendritic length (μm)
M
Number of branches/cell
N
Purkinje cells
Number of branches / Dendritic area
P=0.799
O
MSNs
Number of branches / Total length
P=0.070
P
Purkinje cells
P=0.436
Q
MSNs
P=0.181

Figure S2. Numbers of neurons in the cerebellum and striatum of *Derl1^{NesCre}* and *Derl2^{NesCre}* mice and requirement of Derlin-2 for neurite outgrowth, related to Figure 2

(A and B) Immunohistochemical analysis of cerebellar molecular layer volume using anti-NeuN-stained serial sections from *Derl1^{NesCre}*, *Derl2^{NesCre}*, and control mice aged 37 weeks. (A) Representative immunofluorescence images of the entire cerebellum. (B) Total molecular layer volume was estimated according to Cavalieri's principle from manually measured molecular layer areas of entire cross section of the cerebellum from the anterior (nose) to the posterior (tail) region.

(C and D) Immunohistochemical analysis of cerebellar Purkinje cell number in anti-calbindin antibody-stained sections from *Derl1^{NesCre}*, *Derl2^{NesCre}*, and control mice aged 37 weeks. (C) Representative immunofluorescence images of cerebellar Purkinje cells. (D) Quantification of calbindin-positive Purkinje cell number per 200 μm length of the Purkinje cell layer.

(E and F) Immunohistochemical analysis of the total striatal neuron number using anti-NeuN antibody-stained sections from *Derl1^{NesCre}*, *Derl2^{NesCre}*, and control mice aged 37 weeks. (E) Representative immunofluorescence images of striatal neurons.

(F) Quantification of NeuN-positive cells per unit area.

(G–M) Morphological analysis of Purkinje cells (G–I) and MSNs (J–M) in *Derl2$^{f/f}$* and *Derl2^{NesCre}* mice by Golgi staining at 30 weeks of age. (G) Representative images of Golgi-stained Purkinje cells. (H, I) Quantification of the Purkinje cell dendritic area (H) and the number of branches (I). Ten Purkinje cells were measured in each mouse, and the average from three unrelated mice per genotype are presented. (J) Representative images of Golgi-stained MSNs. (K) Quantification of dendrite complexity by Sholl analysis of Golgi-stained MSNs. The line graph shows the number of dendritic intersections at each indicated distance from the soma. Sholl analysis was performed using the Simple Neurite Tracer and the Sholl analysis plug-in of Fiji software. (L and M) Quantification of total dendritic length (L) and number of branches (M). Ten MSNs were measured from each mouse, and the average from three unrelated mice per genotype is presented.

(N–Q) Quantification of Purkinje cell (N and P) and MSN (O and Q) dendritic complexity as indicated by the number of branches per unit Purkinje dendritic area or total MSN dendritic length.

Data are presented as mean ± SEM. *P < 0.05, **P < 0.01, and ***P < 0.001 by Student's *t*-test (B, D, F, H, I, and L–Q) or repeated measures ANOVA (K). n indicates the number of animals.

Figure S3

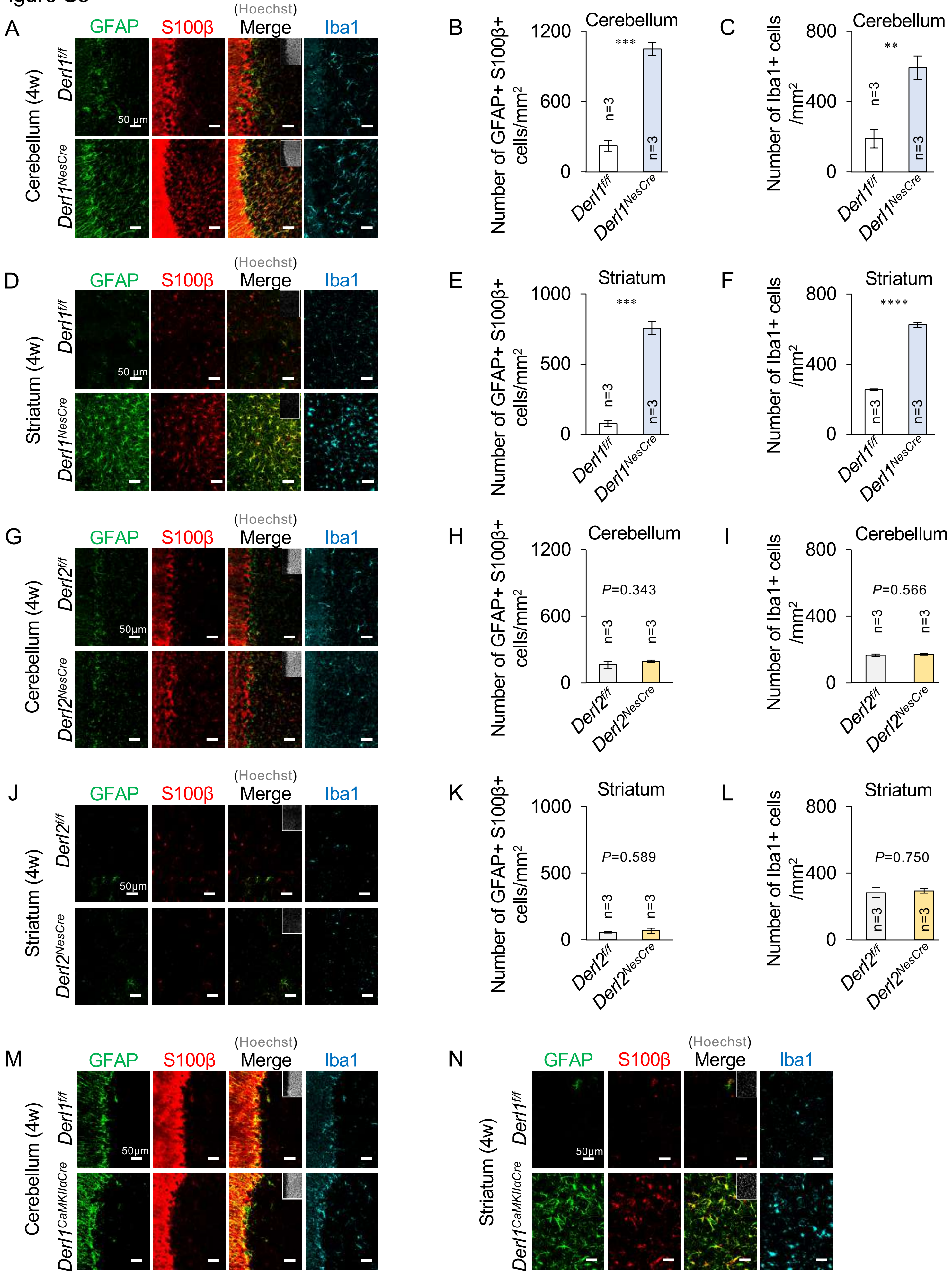

A
GFAP
S100β
(Hoechst)
Merge
Iba1
Cerebellum (4w)
Derl1f/f
Derl1NesCre
50 μm
B
Cerebellum
Number of GFAP+ S100β+ cells/mm2
1200
600
0
***
n=3
n=3
Derl1f/f
Derl1NesCre
C
Cerebellum
Number of Iba1+ cells /mm2
800
400
0
**
n=3
n=3
Derl1f/f
Derl1NesCre
D
Striatum (4w)
50 μm
E
Striatum
1000
500
0
***
F
Striatum
****
G
Cerebellum (4w)
Derl2f/f
Derl2NesCre
50μm
H
Cerebellum
P=0.343
I
Cerebellum
P=0.566
J
Striatum (4w)
K
Striatum
P=0.589
L
Striatum
P=0.750
M
Cerebellum (4w)
Derl1CaMKIIαCre
N
Striatum (4w)

Figure S3. Inflammatory cells in the cerebellum and striatum of *Derl1*^{NesCre}, *Derl2*^{NesCre}, and *Derl1*$^{CaMKII\alpha Cre}$ mice, related to Figure 2

(A–N) Immunohistochemical analysis of astrocytes and microglial cells in cerebellum and striatum by immunostaining for glial fibrillary acidic protein (GFAP, green), S100β (red), and Iba1 (cyan) in sections from 4-week-old *Derl1*^{NesCre}, *Derl2*^{NesCre}, *Derl1*$^{CaMKII\alpha Cre}$, and control mice. Pictures denote as representative immunofluorescence images of the cerebellum (A, G, and M) and striatum (D, J, and N). The numbers of GFAP- and S100β-double positive astrocytes (B, E, H, and K) or Iba1-positive microglia (C, F, I, and L) in the cerebellum and striatum are expressed per mm^2 area.

Data are presented as mean ± SEM. $^{**}P < 0.01$, $^{***}P < 0.001$, and $^{****}P < 0.0001$ by Student's *t*-test. n indicates the number of animals.

Figure S4

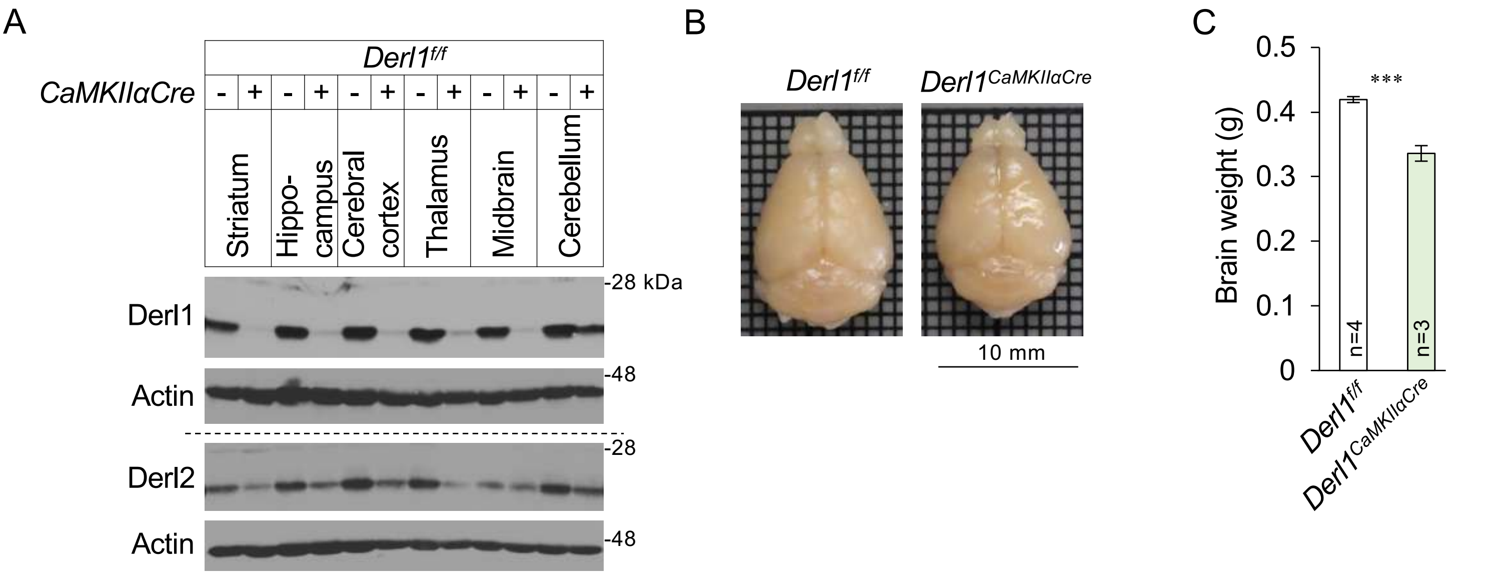

Figure S4. Developmental defects in the brains of neuron-specific Derlin-1 deficient mice, related to Figure 3

(A) Expression of Derlin-1 and Derlin-2 in the brains of *Derl1*$^{f/f}$ and *Derl1*$^{CaMKII\alpha Cre}$ mice at 4 weeks of age. Tissue extracts were analyzed by immunoblotting with the indicated antibodies.

(B) Representative gross brain images of *Derl1*$^{f/f}$ and *Derl1*$^{CaMKII\alpha Cre}$ mice aged 12 weeks.

(C) Comparison of brain weight between *Derl1*$^{f/f}$ and *Derl1*$^{CaMKII\alpha Cre}$ mice aged 12 weeks.

Bar graphs are presented as mean ± SEM. ***P < 0.001 by Student's t-test. n indicates the number of animals.

Figure S5

A

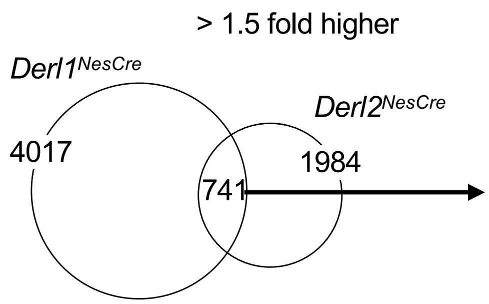

Term	Count	*P* value	Fold enrichment
GO:0034976~response to endoplasmic reticulum stress	13	1.23E-06	6.10
GO:0007155~cell adhesion	33	7.04E-06	2.43
GO:0007411~axon guidance	14	2.90E-04	3.35
GO:0045582~positive regulation of T cell differentiation	6	2.99E-04	9.73
GO:0007010~cytoskeleton organization	11	6.69E-04	3.77
GO:0006935~chemotaxis	11	0.0018	3.32
GO:0030036~actin cytoskeleton organization	12	0.0022	3.01
GO:0007399~nervous system development	22	0.0023	2.08
GO:0000187~activation of MAPK activity	8	0.0053	3.75
GO:0006366~transcription from RNA polymerase II promoter	11	0.0055	2.84

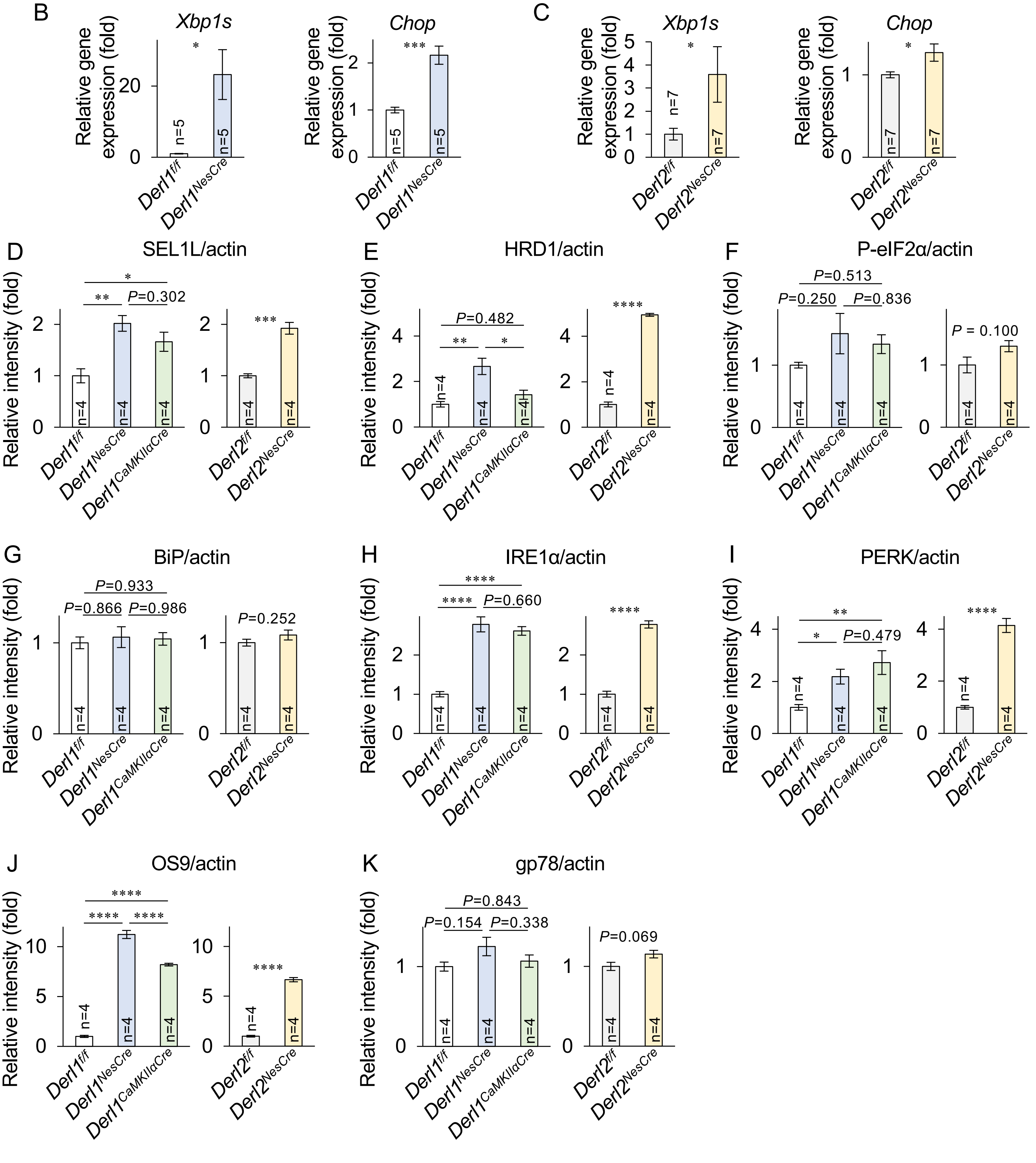

Figure S5. ER stress response in the cerebellum of *Derl1*^{NesCre}, *Derl2*^{NesCre}, and *Derl1*$^{CaMKII\alpha Cre}$ mice, related to Figure 4

(A) GO analysis was performed on 741 genes showing >1.5-fold higher expression in the cerebellum of *Derl1*^{NesCre} and *Derl2*^{NesCre} mice at P28 compared with age-matched *Derl1*$^{f/f}$ and *Derl2*$^{f/f}$ mice. Top ten GO terms in the biological process category are listed. Count indicates the number of enriched genes among the genes categorized by GO annotation.

(B and C) Expression of *Xbp1s* and *Chop* genes in cerebella of *Derl1*^{NesCre} (B) and *Derl2*^{NesCre} (C) mice at 4 weeks of age. Expression levels were estimated by qPCR and normalized to that of *S18*.

(D–K) Expression levels of ER stress-related molecules in the cerebellum at 4–5 weeks of age. Whole tissue lysates from cerebella of *Derl1*$^{f/f}$, *Derl1*^{NesCre}, *Derl1*$^{CaMKII\alpha Cre}$, *Derl2*$^{f/f}$, and *Derl2*^{NesCre} mice were analyzed by IB with the indicated antibodies (Figure 4C). SEL1L, HRD1, P-eIF2α, BiP, IRE1α, PERK, OS9, gp78, and actin band intensities were measured from four unrelated animals per genotype. Amounts of ER stress-related molecules were normalized to those of actin (D–K).

Bar graphs are presented as mean ± SEM. *$P < 0.05$, **$P < 0.01$, ***$P < 0.001$, and ****$P < 0.0001$ by Student's *t*-test or one-way ANOVA. n indicates the number of animals.

Figure S6

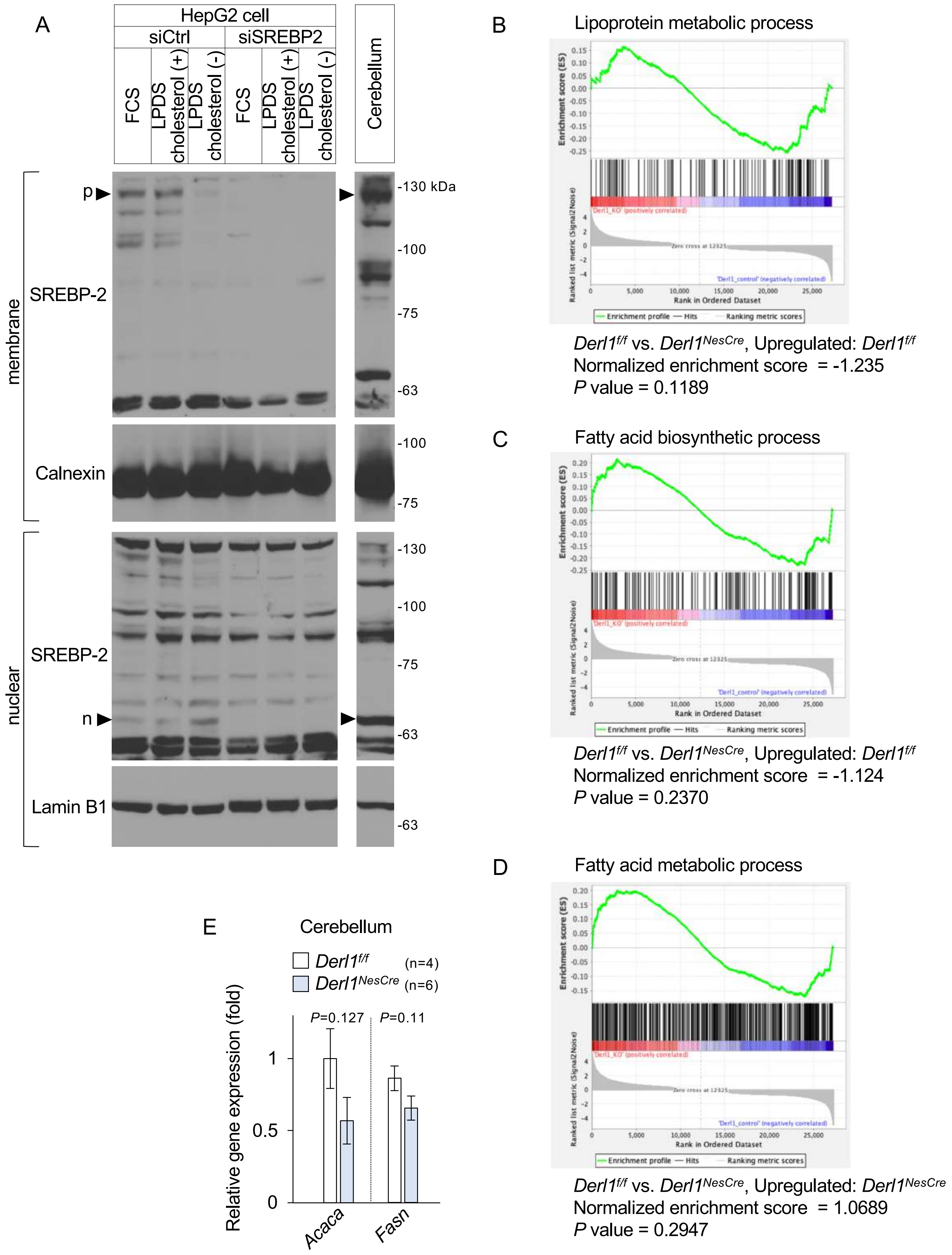

A
HepG2 cell
siCtrl
siSREBP2
FCS
LPDS cholesterol (+)
LPDS cholesterol (-)
Cerebellum
membrane
p
SREBP-2
Calnexin
nuclear
n
Lamin B1
-130 kDa
-100
-75
-63
B
Lipoprotein metabolic process
Derl1f/f vs. Derl1NesCre, Upregulated: Derl1f/f
Normalized enrichment score = -1.235
P value = 0.1189
C
Fatty acid biosynthetic process
Derl1f/f vs. Derl1NesCre, Upregulated: Derl1f/f
Normalized enrichment score = -1.124
P value = 0.2370
D
Fatty acid metabolic process
Derl1f/f vs. Derl1NesCre, Upregulated: Derl1NesCre
Normalized enrichment score = 1.0689
P value = 0.2947
Enrichment score (ES)
Ranked list metric (Signal2Noise)
Rank in Ordered Dataset
Enrichment profile
Hits
Ranking metric scores
E
Cerebellum
Derl1f/f (n=4)
Derl1NesCre (n=6)
P=0.127
P=0.11
Relative gene expression (fold)
1
0.5
0
Acaca
Fasn

Figure S6. Alterations in fatty acid and lipoprotein metabolic process-related gene expression in the cerebella of Derlin-1-deficient mice, related Figure 5

(A) Validation of precursor SREBP-2 and nuclear SREBP-2 measurements by comparing IB results using HepG2 cell lysates and P28 cerebellar lysates. HepG2 cells were transfected as indicated with siRNA in the presence or absence of 10 μg/mL cholesterol and 5% lipoprotein-deficient serum (LPDS) in the medium. After incubation for 16–20 h, cells were treated with 25 μg/mL of the cysteine proteinase inhibitor ALLN and harvested after 2–4 h. The membrane and nuclear fractions from HepG2 cell and cerebellar lysates were analyzed by IB using the indicated antibodies. A protein band in the membrane fraction (precursors SREBP-2, p) was reduced and a 68-kDa band in the nuclear fraction (nuclear SREBP-2, n) was increased by cholesterol depletion from the culture medium of cells transfected with control siRNA (siCtrl) cells but not cells transfected with SREBP-2 siRNA (siSREBP-2). A similar 125-kDa band (p) in the membrane fraction and a 68-kDa band in the nuclear fraction (n) were also observed in cerebellar extracts. siCtrl, siRNA for control; siSREBP-2, siRNA for SREBP-2; p, pSREBP-2; n, nSREBP-2.

(B–D) Gene set enrichment analysis (GSEA) showing differential expression of 128 genes related to the GO term “Lipoprotein metabolic process” (A), 146 genes related to the GO term “Fatty acid biosynthetic process” (B) and 416 genes related to the GO term “Fatty acid metabolic process.” GSEA shows gene expression changes in the cerebellum of *Derl1*^{NesCre} mice relative to control mice. The Enrichment plot shows the distribution of genes in each set that are positively (red) or negatively (blue) correlated with Derlin-1 deficiency.

(E) Expression of fatty acid biosynthesis-related genes *Acaca* and *Fasn* in the cerebella of *Derl1*$^{f/f}$ and *Derl1*^{NesCre} mice at P28. Gene expression levels were estimated by qPCR and normalized to that of *S18*.

Bar graphs are presented as mean ± SEM. *P* value by Student’s *t*-test. n indicates the number of animals.

Figure S7

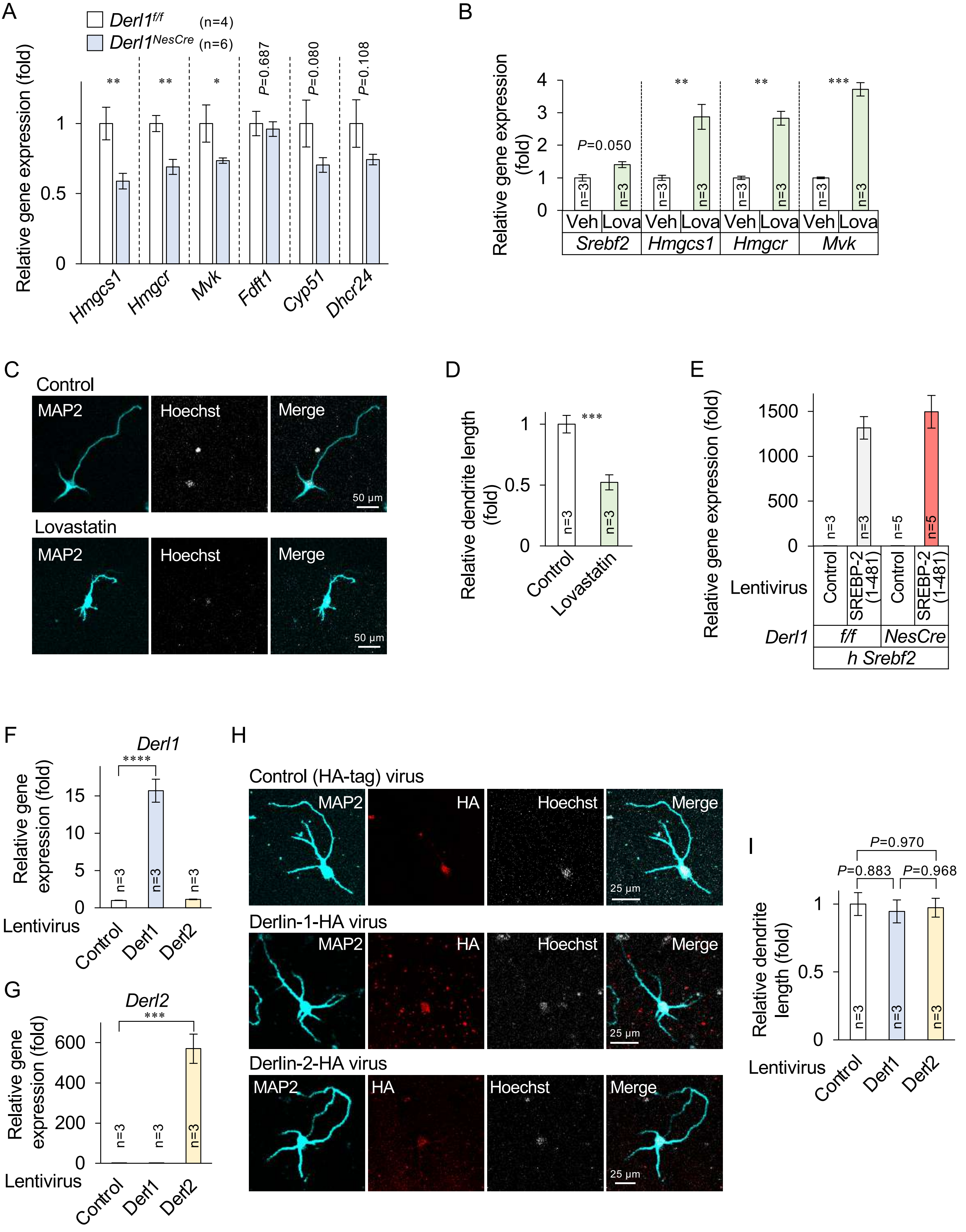
A
Derl1f/f (n=4)
Derl1NesCre (n=6)
Relative gene expression (fold)
**
**
*
P=0.687
P=0.080
P=0.108
Hmgcs1
Hmgcr
Mvk
Fdft1
Cyp51
Dhcr24
B
Relative gene expression (fold)
P=0.050
**
**
***
Veh
Lova
Srebf2
Hmgcs1
Hmgcr
Mvk
n=3
C
Control
Lovastatin
MAP2
Hoechst
Merge
50 µm
D
Relative dendrite length (fold)
***
n=3
Control
Lovastatin
E
Relative gene expression (fold)
n=3
n=5
Lentivirus
Control
SREBP-2 (1-481)
Derl1
f/f
NesCre
h Srebf2
F
Derl1
Relative gene expression (fold)
****
n=3
Lentivirus
Control
Derl1
Derl2
G
Derl2
Relative gene expression (fold)
***
n=3
Lentivirus
Control
Derl1
Derl2
H
Control (HA-tag) virus
Derlin-1-HA virus
Derlin-2-HA virus
MAP2
HA
Hoechst
Merge
25 µm
I
P=0.970
P=0.883
P=0.968
Relative dendrite length (fold)
n=3
Lentivirus
Control
Derl1
Derl2

Figure S7. Requirement of Derlin-1 for the cholesterol biosynthetic pathway, related to Figure 6.

(A) Expression of cholesterol biosynthesis-related genes in 3 DIV cortical neurons derived from $Derl1^{f/f}$ and $Derl1^{NesCre}$ embryos.

(B–D) Requirement of cholesterol biosynthesis for neurite outgrowth. Cortical neurons derived from wild-type C57BL/6 embryos were treated with vehicle or 1 μM lovastatin and cultured for three days. Gene expression levels of cholesterol biosynthesis-related genes in neurons were estimated by qPCR and normalized to that of *S18* (B). Neurons were stained with anti-MAP2 antibody (C). Dendritic length of MAP2-positive neuron was quantified using ImageJ software (D). One hundred neurons were measured in each culture dish and averaged to obtain n = 1.

(E) Expression of *human Srebf2* in 3 DIV cortical neurons derived from $Derl1^{f/f}$ and $Derl1^{NesCre}$ embryos. Cortical neurons were infected with lentivirus expressing control (pRRL-Venus-HA) or SREBP-2 (1–481) [pRRL-Venus-hSREBP-2(1–481)-HA] and cultured for three days.

(F–I) No effect of exogenous Derlin-1 and Derlin-2 for neurite outgrowth in cultured neurons. Expression of *Derl1* (F) and *Derl2* (G) mRNAs in cortical neurons and representative immunofluorescence images of MAP2- and HA-positive cortical neurons (H). Cortical neurons were infected with lentivirus expressing control (Venus-HA), mDerlin-1-HA, and mDerlin-2-HA and cultured for three days. Dendritic length was quantified following MAP2 staining (I). One hundred neurons were measured in each culture dish and averaged to obtain n = 1. Gene expression levels were estimated by qPCR and normalized to that of *S18*.

Bar graphs are presented as mean ± SEM. *P < 0.05, **P < 0.01, ***P < 0.001, and ****P < 0.0001 by Student's *t*-test (A, B, and D) or one-way ANOVA (F, G, and I). n indicates the number of independent dishes.
