# Supplementary material for: ERAD components Derlin-1 and Derlin-2 are essential for postnatal brain development and motor function
Source: iScience. 2021 Jun 19;24(7):102758. doi: 10.1016/j.isci.2021.102758 (PMC8324814; doi:10.1016/j.isci.2021.102758)
Supplement: Table S3. Primers for quantitative real-time PCR, Related to key resources table [file mmc4.docx]

**Table S3. Primers for quantitative real-time PCR, related to KEY RESOURCES TABLE**

| Species | Gene name | Forward | Reverse | Reference |
| --- | --- | --- | --- | --- |
| Mouse | Xbp1s | GGTCTGCTGAGTCCGCAGCAGG | GGTCTGCTGAGTCCGCAGCAGG | (Liu et al., 2016) |
| Mouse | Chop | CCACCACACCTGAAAGCAGAA | AGGTGAAAGGCAGGGACTCA | (Liu et al., 2016) |
| Mouse | Hmgcs1 | AAATGCCAGACCTACAGGTGG | ATGCTGCATGTGTGTCCCA | Harvard Primer Bank ID: 148747293c3 |
| Mouse | Mvk | TGACCTCCATTGACGCAATATC | CCACCCCGAGAGCATTCAG | Harvard Primer Bank ID: 31980640c3 |
| Mouse | Fdft1 | AGAAGGACCGACAAGTGCTG | CCCAGTCCTGTTTGGAGGTC | N/A^#)^ |
| Mouse | Cyp51 | AACGAAGACCTGAATGCAGAAG | GTGGGCTATGTTAAGGCCACT | Harvard Primer Bank ID: 71061450c3 |
| Mouse | Dhcr24 | CGCTGCGAGTCGGAAAGTA | GTCACCTGACCCATAGACACC | Harvard Primer Bank ID: 114155128c1 |
| Mouse | Hmgcr | AGAGCGAGTGCATTAGCAAAG | GATTGCCATTCCACGAGCTAT | Harvard Primer Bank ID: 160358777c2 |
| Mouse | s18 | TCCAGCACATTTTGCGAGTA | CAGTGATGGCGAAGGCTATT | (Hattori et al., 2016) |
| Mouse | Srebf2 | TGGACCTCACGGGGGACTC | CTCTCCCACTTGATTGCTGACA | N/A^#)^ |
| Human | Srebf2 | CGAATTGAAAGACCTGGTCATG | TCCTCAGAACGCCAGACTTGT | (Chittur et al., 2008) |
| Mouse | Acaca | AATGAACGTGCAATCCGATTTG | ACTCCACATTTGCGTAATTGTTG | Harvard Primer Bank ID: 125656172c3 |
| Mouse | Fasn | AGGTGGTGATAGCCGGTATGT | TGGGTAATCCATAGAGCCCAG | Harvard Primer Bank ID: 93102408c1 |
| Mouse | Derl1 | ATCAGCCCGGCCTACTTCTT | GGCCCACGGGGAAGTAAAAG | N/A^#)^ |
| Mouse | Derl2 | GGTGCTCATGGGGTTTTCCC | TATTCCACCAGGCTGATTGGG | N/A^#)^ |
| #) Forward and reverse primers were newly designed by the authors with NCBI Primer-BLAST. | | | | |
